# Evaluating lesion-specific preprocessing pipelines for rs-fMRI in stroke patients: Impact on functional connectivity and behavioral prediction

**DOI:** 10.1162/IMAG.a.6

**Published:** 2025-05-30

**Authors:** Alix Lamouroux, Julie Coloigner, Pierre Maurel, Nicolas Farrugia, Giulia Lioi

**Affiliations:** IMT Atlantique, Lab-STICC UMR CNRS 6285 F-29238, Brest, France; Univ Rennes, Inria, CNRS, Inserm, Rennes, France

**Keywords:** fMRI preprocessing, functional connectivity, stroke

## Abstract

Functional magnetic resonance imaging (fMRI) is essential for studying brain function and connectivity. Resting-state fMRI, which captures spontaneous brain activity without task requirements, is particularly suited for individuals with post-stroke impairments. However, the inherent noise and artifacts in fMRI signals can compromise analysis accuracy, especially in stroke patients with complex neurological conditions. Currently, there is no consensus on the best preprocessing approach for stroke fMRI data. In this study, we design and evaluate three preprocessing pipelines: a standard pipeline, an enhanced pipeline that accounts for lesions when computing tissue masks, and a stroke-specific pipeline that incorporates independent component analysis to address lesion-driven artifacts. These pipelines are assessed for their effectiveness in reducing spurious connectivity and improving the prediction of behavioral outcomes on a large stroke dataset. Using metrics such as connectivity mean strength and functional connectivity contrast, our results indicate that the stroke-specific pipeline significantly reduces spurious connectivity without impacting behavioral predictions. These findings underscore the need for tailored preprocessing strategies in stroke fMRI research to enhance the reliability and accuracy of connectivity measures. In addition, we make the stroke-specific pipeline accessible by designing an open-source tool (fMRIStroke), in order to ensure replicability of our results and to contribute to best practices.

## 1 Introduction

Functional magnetic resonance imaging (fMRI) is a valuable tool in neuroscience, providing detailed insights into brain activity and connectivity. Resting-state fMRI (rs-fMRI), in particular, captures spontaneous brain activity without the need for the subject to be engaged in a specific task, making it ideal for individuals with neurological conditions such as stroke, where task performance might be impaired. Unlike task-based fMRI, which relies on specific activities to stimulate brain regions, rs-fMRI evaluates intrinsic brain functions (Fox et al., 2005), allowing researchers to detect disruptions or abnormalities in neural activity, offering valuable insights into various neurological conditions (Ovadia-Caro et al., 2014).

One significant use of rs-fMRI is studying functional connectivity (FC), which examines the temporal correlations between spatially distant brain regions. Understanding these interactions allows researchers to explore how different brain regions form networks (Bassett & Sporns, 2017), thereby enhancing our knowledge of both healthy and pathological brain states (Fornito et al., 2015). In particular, FC studies have been numerous in stroke research, providing insights into how strokes disrupt and reorganize brain networks (Carter et al., 2012;Grefkes & Fink, 2014;Ktena et al., 2019;Park et al., 2011). For instance,Ktena et al. (2019)demonstrated that analyzing FC can predict functional outcomes in stroke patients, enabling clinicians to better anticipate recovery trajectories. Additionally,Grefkes and Fink (2014)highlighted that examining FC patterns helps understand the role of different brain areas in recovery and the varying effects of treatments, which is crucial for developing personalized rehabilitation strategies. The reliability of FC measurements, however, is highly dependent on the preprocessing of fMRI data. Standard preprocessing steps—motion correction, spatial normalization, and denoising—are crucial for mitigating artifacts such as head motion and physiological noise in healthy subjects. In stroke patients, these processes become even more important due to the unique challenges posed by cardiovascular pathologies (Siegel et al., 2017).

A fundamental assumption of most rs-fMRI research is that neurovascular coupling—the relationship between neural activity and blood flow—is relatively consistent across brain areas, time, and individuals. While this assumption generally holds in healthy populations and allows for reliable mapping of spatial and temporal brain relationships (Buckner et al., 2013), it can become problematic in the context of cerebrovascular diseases such as stroke (Carusone et al., 2002;Pineiro et al., 2002). Hemodynamic lag, which refers to the delayed arrival of the blood-oxygen-level-dependent (BOLD) signal in some brain regions, is often observed in stroke patients (Siegel, Snyder, et al., 2016). These lags can systematically distort FC measurements, leading to inaccurate interpretations of brain connectivity (Christen et al., 2015;Siegel et al., 2017;Siegel, Snyder, et al., 2016). In particular, these delays can alter the temporal alignment of BOLD signals between regions, leading to an underestimation of true connectivity or the introduction of spurious correlations. Thus,Siegel et al. (2017)emphasize the need for additional preprocessing steps to empirically identify and control for these effects.

Moreover, the complexity of stroke pathology, characterized by heterogeneous lesion locations and sizes, introduces artifacts into fMRI data, impacting the quality of standard preprocessing steps. For example, stroke-induced brain tissue displacement can affect the accuracy of registration and normalization processes, making the incorporation of lesion masks during normalization recommended (Brett et al., 2001). Additionally,Yourganov et al. (2017)found that the BOLD signals within lesion areas might show strong correlations despite the tissue being non-functional. This makes it difficult to distinguish between real FC and spurious correlations caused by the lesion. To address this, they proposed an independent component analysis (ICA)-based approach to identify and remove lesion-related noise components, thereby improving the reliability of FC measures in stroke patients.

Despite the importance of preprocessing, there is no consensus on the best approach to preprocess stroke fMRI data for FC analysis. In this study, we address this gap by comparing different preprocessing pipelines for fMRI data in stroke patients, focusing on their effectiveness in minimizing spurious connectivity and improving the prediction of behavioral outcomes. We employ three distinct pipelines: CompCorGS, which includes traditional confounding time series (Behzadi et al., 2007); CompCorLesionGS, which incorporates lesion masks; and ICLesionCompCorGS, which adds ICA-based lesion regressors (Yourganov et al., 2017). Our goal is to determine which pipeline most effectively mitigates spurious connectivity, using metrics such as positive mean connectivity strength, negative mean strength, and functional connectivity contrast (FCC). Additionally, we assess the impact of each pipeline on predicting behavioral scores across multiple domains, including language, motor, visual, and memory functions. To ensure replicability and transparency, we utilize the open-source tool fMRIprep for standard preprocessing and develop a novel open-source tool (fMRIStroke), specifically designed to preprocess stroke fMRI data with additional steps. These tools facilitate standardized preprocessing steps (Esteban, Markiewicz, et al., 2018) and enable other researchers to replicate and validate our findings.

## 2 Materials and Methods

### 2.1 Dataset

The dataset used is the public dataset presented inCorbetta et al. (2015). Written informed consent was obtained from all participants in accordance with the Declaration of Helsinki ND procedures established by the Washington University in Saint Louis Institutional Review Board. All participants were compensated for their time. All aspects of this study were approved by the Washington University School of Medicine (WUSM) Internal Review Board.

First-time stroke patients (n = 132) were recruited through the in-patient service at Barnes-Jewish Hospital and the Rehabilitation Institute of St. Louis (mean age 52.8 years with range 22–77; 119 right handed; 63 females; 64 right hemispheres). The dataset comprises patients with both ischemic and hemorrhagic strokes. Detailed inclusion and exclusion criteria are provided in the original paper. Patients were seen 2 weeks, 3 months, and 1 year poststroke onset.

**Behavioral assessment**Participants underwent a neuropsychological battery to assess performance across multiple behavioral domains, including attention, visual memory, verbal memory, language, motor, and visual functions, seeSiegel, Ramsey, et al. (2016)for more details. Scores from these assessments were used as target variables in subsequent predictive modeling.

#### 2.1.1 MRI procedure

All imaging were performed using a Siemens 3T Tim-Trio scanner at the Washington University School of Medicine (WUSM) and the standard12-channel head coil. The MRI protocol included structural and functional scans. Structural scans included a sagittal T1-weighted MP-RAGE (TR=1950ms,TE=2.26s, flip angle = 90°, voxel size=1.0×1.0×1.0mm). Resting-state functional scans were acquired with a gradient echo EPI sequence (TR=2s,TE=27ms,32contiguous 4 mm slices,4×4mm in-plane resolution) during which participants were instructed to fixate on a small cross. Six to eight resting-state fMRI (rsfMRI) runs, each including 128 volumes (30 min total), were acquired. Participants with low imaging quality and less than 5 min of retained rsfMRI data after strict motion scrubbing were excluded from further analysis. The fraction of participants providing useful fMRI data was 105 of 132 (91 ischemic, 14 hemorrhagic) patients at 2 weeks, 89 of 103 (75 ischemic, 14 hemorrhagic) patients at 3 months, 76 of 88 (65 ischemic, 11 hemorrhagic) patients at 1 year based on previous studies (Siegel, Ramsey, et al., 2016);Siegel et al., 2018). The full MRI protocol is described inCorbetta et al. (2015).

#### 2.1.2 Lesion analysis

Lesion segmentation masks were provided with the dataset and were performed as described inCorbetta et al. (2015). Lesions were manually segmented on individual structural MRIs (T1-weighted MP-RAGE, T2-weighted spin-echo images, and FLAIR images) using the Analyze biomedical imaging software system (www.mayoclinic.org;Robb & Hanson, 1991). Two board-certified neurologists (Drs Maurizio Corbetta and Alex Carter) reviewed all segmentations. SeeSupplementary Figure S3for lesion map.

### 2.2 MRI preprocessing

#### 2.2.1 Anatomical MRI preprocessing

Results included in this manuscript come from preprocessing performed using*fMRIPrep*23.0.0 (Esteban, Blair, et al., 2018;Esteban, Markiewicz, et al., 2018; RRID:SCR_016216), which is based on*Nipype*1.8.5 (K. Gorgolewski et al., 2011;K. J. Gorgolewski et al., 2018; RRID:SCR_002502). The T1-weighted (T1w) image was corrected for intensity non-uniformity with N4BiasFieldCorrection (Tustison et al., 2010), distributed with ANTs 2.3.3 (Avants et al., 2008; RRID:SCR_004757), and used as T1w-reference throughout the workflow. The T1w-reference was then skull stripped with a*Nipype*implementation of the antsBrainExtraction.sh workflow (from ANTs), using OASIS30ANTs as target template. Brain tissue segmentation of cerebrospinal fluid (CSF), white matter (WM), and gray matter (GM) was performed on the brain-extracted T1w using fast (FSL 6.0.5.1:57b01774, RRID:SCR_002823;Zhang et al., 2001). Brain surfaces were reconstructed using recon-all (FreeSurfer 7.3.2, RRID:SCR_001847;Dale et al., 1999), and the brain mask estimated previously was refined with a custom variation of the method to reconcile ANTs-derived and FreeSurfer-derived segmentations of the cortical gray matter of Mindboggle (RRID:SCR_002438;Klein et al., 2017). Volume-based spatial normalization to one standard space (MNI152NLin2009cAsym) was performed through nonlinear registration with antsRegistration (ANTs 2.3.3) with cost function masking with the lesion mask to minimize warping of healthy tissue into damaged areas (Brett et al., 2001), using brain-extracted versions of both T1w reference and the T1w template. The following template was selected for spatial normalization:*ICBM 152 Nonlinear Asymmetrical template version 2009c*(Fonov et al., 2009; RRID:SCR_008796; TemplateFlow ID: MNI152NLin2009cAsym).

#### 2.2.2 Functional MRI preprocessing

The preprocessing steps of the functional MRIs are described inFigure 1. fMRIprep was used to preprocess the rsfMRI data and fMRIstroke was then used to add stroke-specific steps into the preprocessing pipeline, perform denoising, and compute connectivity matrices as described below.

**Fig. 1.**
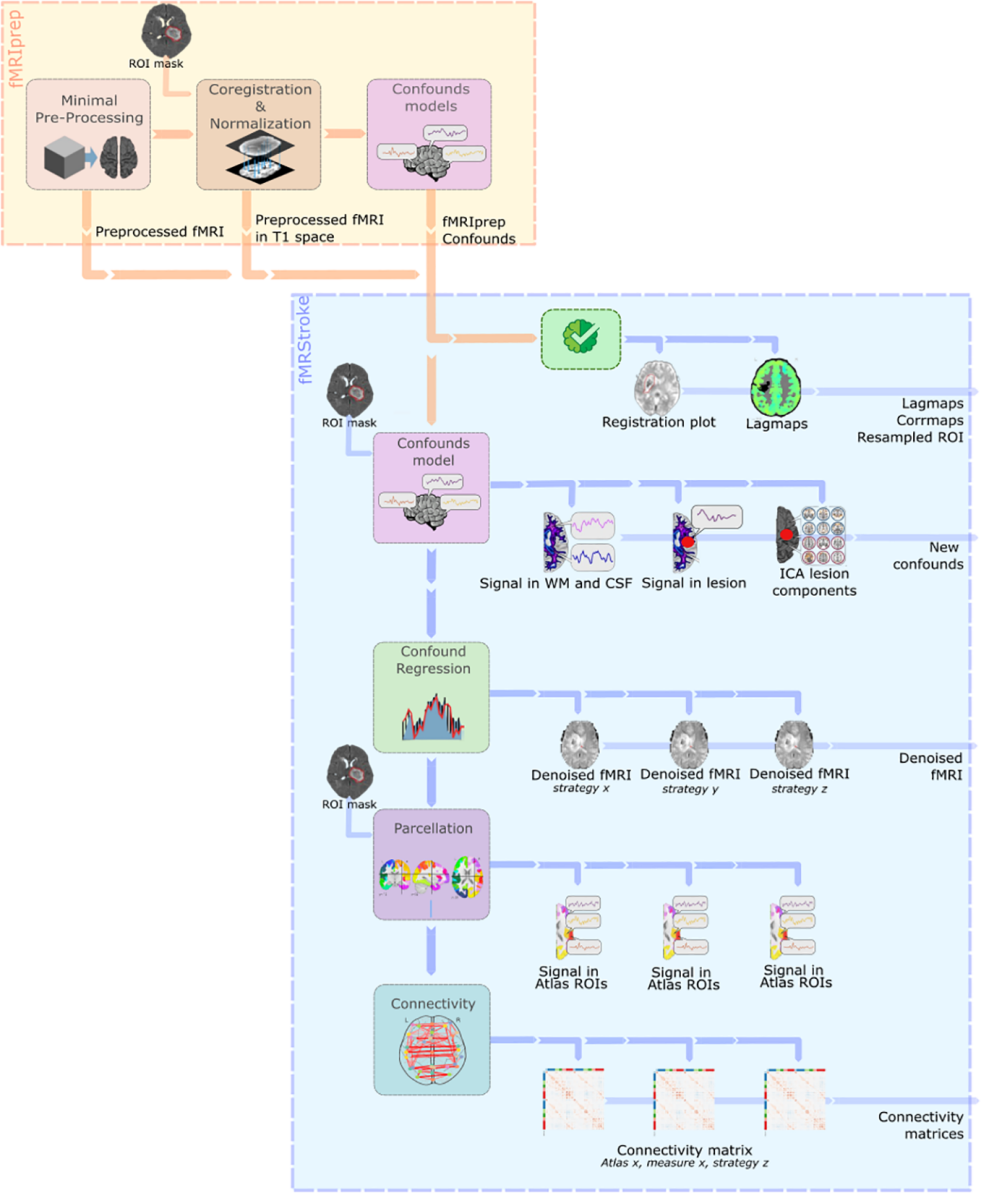
Preprocessing and connectivity pipeline. First in the yellow frame, fMRIprep is used to preprocess the rsfMRI data. Minimal preprocessing included (1) generation of a reference volume and its skull-stripped version, (2) estimation of the head motion parameters with respect to the BOLD reference (transformation matrices, and six corresponding rotation and translation parameters), (3) slice-timing correction of the BOLD runs. The BOLD reference was then co-registered to the T1w reference and resampled into standard space. Finally, several confounding time series were calculated based on the preprocessed BOLD. Next, fMRIStroke in the blue frame is run on the fMRIprep derivatives, adding stroke-specific quality checks such as hemodynamic lag computation and stroke-specific confounding time-series estimation. Finally, the rsfMRI data are denoised using selected confounding time series, and the connectivity matrices are computed.

**fMRIprep**The following text was automatically generated by*fMRIPrep*23.0.0. First, a reference volume and its skull-stripped version were generated using a custom methodology of*fMRIPrep*. Head motion parameters with respect to the BOLD reference (transformation matrices, and six corresponding rotation and translation parameters) are estimated before any spatiotemporal filtering using mcflirt (FSL 6.0.5.1:57b01774;Jenkinson et al., 2002). BOLD runs were slice-time corrected to 0.782 s (0.5 of slice acquisition range 0–1.56 s) using 3dTshift from AFNI (Cox & Hyde, 1997; RRID:SCR_005927). The BOLD time series (including slice-timing correction when applied) were resampled onto their original, native space by applying the transforms to correct for head motion. These resampled BOLD time series will be referred to as*preprocessed BOLD in original space*, or just*preprocessed BOLD*. The BOLD reference was then co-registered to the T1w reference using bbregister (FreeSurfer) which implements boundary-based registration (Greve & Fischl, 2009). Co-registration was configured with six degrees of freedom. Several confounding time series were calculated based on the*preprocessed BOLD*: framewise displacement (FD), DVARS, and three region-wise global signals. FD was computed using two formulations following Power (absolute sum of relative motions;Power et al., 2014) and Jenkinson (relative root mean square displacement between affines;Jenkinson et al., 2002). FD and DVARS are calculated for each functional run, both using their implementations in*Nipype*(following the definitions byPower et al., 2014). The three global signals are extracted within the CSF, the WM, and the whole-brain masks. Additionally, a set of physiological regressors were extracted to allow for component-based noise correction (*CompCor*;Behzadi et al., 2007). Principal components are estimated after high-pass filtering the*preprocessed BOLD*time series (using a discrete cosine filter with 128 s cutoff) for the two*CompCor*variants: temporal (tCompCor) and anatomical (aCompCor). tCompCor components are then calculated from the top 2% variable voxels within the brain mask. For aCompCor, three probabilistic masks (CSF, WM, and combined CSF+WM) are generated in anatomical space. The implementation differs from that of Behzadi et al. in that instead of eroding the masks by two pixels on BOLD space, a mask of pixels that likely contain a volume fraction of GM is subtracted from the aCompCor masks. This mask is obtained by dilating a GM mask extracted from the FreeSurfer’s*aseg*segmentation, and it ensures components are not extracted from voxels containing a minimal fraction of GM. Finally, these masks are resampled into BOLD space and binarized by thresholding at 0.99 (as in the original implementation). Components are also calculated separately within the WM and CSF masks. For each CompCor decomposition, the*k*components with the largest singular values are retained, such that the retained components’ time series are sufficient to explain 50% of variance across the nuisance mask (CSF, WM, combined, or temporal). The remaining components are dropped from consideration. The head motion estimates calculated in the correction step were also placed within the corresponding confounds file. The confound time series derived from head motion estimates and global signals were expanded with the inclusion of temporal derivatives and quadratic terms for each (Satterthwaite et al., 2013). Frames that exceeded a threshold of 0.5 mm FD or 1.5 standardized DVARS were annotated as motion outliers. Additional nuisance time series are calculated by means of principal components analysis of the signal found within a thin band (*crown*) of voxels around the edge of the brain, as proposed byPatriat et al. (2017). The BOLD time series were resampled into standard space, generating a*preprocessed BOLD run in MNI152NLin2009cAsym space*. First, a reference volume and its skull-stripped version were generated using a custom methodology of*fMRIPrep*. All resamplings can be performed with*a single interpolation step*by composing all the pertinent transformations (i.e., head motion transform matrices, susceptibility distortion correction when available, and co-registrations to anatomical and output spaces). Gridded (volumetric) resamplings were performed using antsApplyTransforms (ANTs), configured with Lanczos interpolation to minimize the smoothing effects of other kernels (Lanczos, 1964). Non-gridded (surface) resamplings were performed using mri_vol2surf (FreeSurfer).

Many internal operations of*fMRIPrep*use*Nilearn*0.9.1 (Abraham et al., 2014; RRID:SCR_001362), mostly within the functional processing workflow. For more details of the pipeline, see the section corresponding to workflows in*fMRIPrep*’s documentation.

**fMRIStroke**Additional “stroke specific” confounding time series were computed using the fMRIStroke libraryLamouroux et al. (2024)(https://github.com/alixlam/fmristroke) as displayed inFigure 1in the blue panel. Region-wise average signal excluding lesion mask, region-wise average signal in lesion mask and region-wise average signal including lesion mask, and finally a set of lesion-related regressors are computed following the methods proposed byYourganov et al. (2017). Independent components are calculated on the BOLD signal and components that overlap more than≥ 5%with the lesion mask (unlikely to include signal related to neuronal activity) are identified as potential noise components. The remaining components are dropped from consideration. If no component met this criterion, ICA-based regressors were not included in the model.

Additional quality checks included plots of lesion masks in T1w and standard space to visually ensure registration and normalization accuracy and the computation of a hemodynamic lagmap (Siegel et al., 2017). To define these lagmaps, rsfMRI data were first temporal bandpass filtered, retaining frequencies between 0.009 and 0.09 Hz. Next, a reference signal was generated from the average time course in each subject’s gray matter compartment (excluding voxels in lesion masks). Lagged cross-correlation analysis with reference to the global gray matter reference signal was performed for each voxel over the range± 4TRs (± 8s). The lag was defined as the shift that maximizes the cross-correlation function. FollowingSiegel et al.’s (2017)recommendations, patients with a mean lag in the affected hemisphere exceeding 1 s are excluded from the analysis. This exclusion is due to the fact that severe and widespread lags result in FC data that might be too altered to be usable in the analysis.

**Fig. 2.**
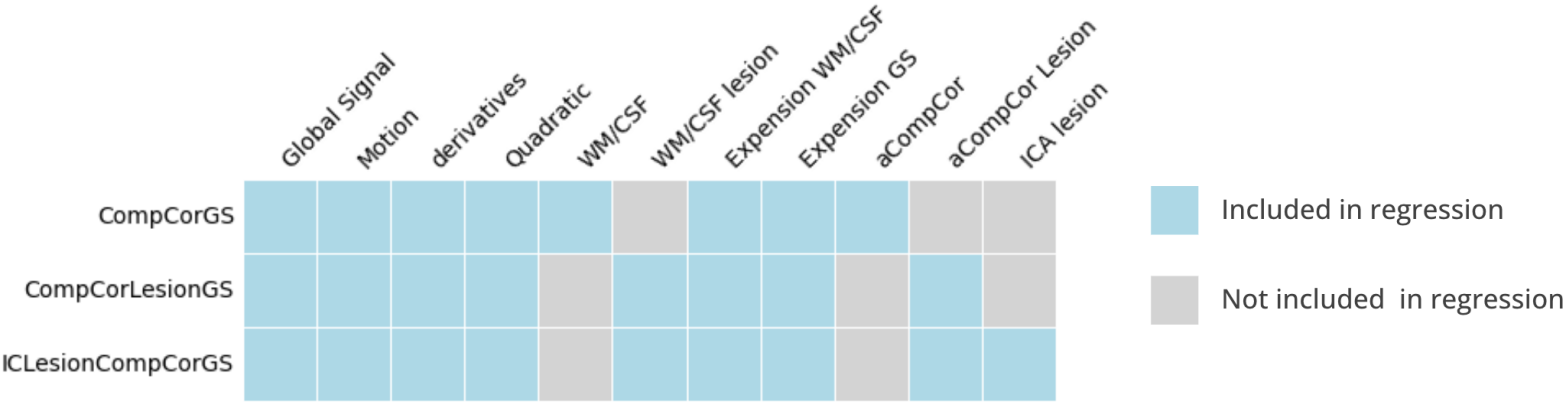
Denoising confound regressors in the three tested pipelines: CompCorGS includes typical confounding time series including CompCor regressorsBehzadi et al. (2007), CompCorLesionGS an adaptation of CompCorGS including lesion masks when computing tissue masks, and ICLesionCompCorGS which is a further extension of CompCorLesionGS with ICA lesion-based regressors. GS: global signal, WM: white matter, CSF: cerebrospinal fluid, expansion: quadratic terms and derivatives

Three pipelines differing by the confounding time series included as regressors for denoising were considered (Fig. 2). CompCorGS included typical confounding time series (Behzadi et al., 2007): global signal (mean signal in the whole brain), motion estimates and derivatives, tissue signals (WM and CSF), and anatomical compcor regressors. CompCorLesionGS was an adaptation of the latter but taking into account lesion masks when computing tissue masks, including the mask in the CSF mask and excluding it from other tissue masks. Finally ICLesionCompCorGS added new regressors to CompCorLesionGS based on the ICA performed by fmriStroke as described above, following the method presented inYourganov et al. (2017).

### 2.3 Connectivity

FC from resting-state fMRI data was computed using Nilearn with the Schaefer Atlas (400 regions) (Schaefer et al., 2018). The brain was parcellated and the average BOLD signal time series was extracted for each region. Pearson’s correlation with the Ledoit-Wolf shrinkage covariance estimator (Honnorat & Habes, 2022;Ledoit & Wolf, 2004) was used to measure functional connectivity, resulting in a400×400matrix where each element represents the correlation between the time series of two regions.

### 2.4 Pipeline evaluation

#### 2.4.1 Metrics

To evaluate the effectiveness of different preprocessing pipelines, we define three key metrics: positive mean strength, negative mean strength, and functional connectivity contrast (FCC). These measures are designed to assess both the reduction of spurious connectivity and the preservation of meaningful functional signals, with a particular focus on the regions overlapping with stroke lesions. By jointly considering these metrics, we aim to ensure that artifact removal does not come at the cost of eliminating relevant neural activity.

**Positive and Negative Mean Strength**Positive mean strength measures the mean connectivity strength of regions overlapping with the stroke lesion to the rest of brain using only positive correlation values. Connectivity strength of a region of interest (ROI) is the sum of all its edges, representing the degree of correlation between the ROI and other brain regions. Given that stroke lesions typically comprise non-functional, dead tissue, we hypothesize that these regions should exhibit minimal connectivity with other brain areas (Yourganov et al., 2017). Hence this metric assesses whether a preprocessing pipeline successfully minimizes spurious connectivity of the lesion site with the rest of the brain. Negative mean strength is similarly computed using only negative correlation values. Although negative correlations are more challenging to interpret, we hypothesize that these correlations should also be minimal.

**FCC**The particularity of the Schaefer atlas is that the 400 regions can be grouped into functional networks, specifically Yeo networks (Thomas Yeo et al., 2011). FCC is then designed to evaluate the distinction between within-network edges (WNE) and between-network edges (BNE). It is based on the hypothesis that a preprocessing pipeline improving signal-to-noise ratio should enhance this distinction. FCC is defined as the Z-statistic from the Wilcoxon rank-sum test comparing WNE and BNE correlation values, with higher FCC indicating greater distinction between WNEs and BNEs (Kassinopoulos & Mitsis, 2022). In addition to measuring network structure, FCC can also serve as an indicator of meaningful signal loss during preprocessing. If a preprocessing pipeline disproportionately removes functional signal along with noise, it may reduce the separation between within-network and between-network connectivity, thereby lowering FCC values. Conversely, if artifact removal effectively preserves meaningful connectivity patterns, FCC should remain stable or improve. By comparing FCC across preprocessing pipelines, we can assess whether stroke-specific pipelines successfully reduce lesion-related artifacts without disrupting functional network integrity.

**Q value**In addition to the primary metrics described above, we computed the Q-value metric (Ciric et al., 2017) to further explore whether preprocessing pipelines impact the modular organization of functional connectivity networks. Unlike FCC, which evaluates predefined network integrity, Q-values provide a data-driven measure of how well functional networks self-organize into distinct modules. However, a decrease in Q-value can be interpreted in multiple ways. In healthy individuals, a reduction in Q-value can typically be associated with a loss of meaningful signalCiric et al. (2017). In stroke patients, this interpretation is less straightforward, as a decrease may also reflect the successful removal of lesion-driven spurious connections that artificially inflated modularity estimates. Given this ambiguity, we opted to keep Q-value as a complementary analysis rather than a primary evaluation metric. The full results of this analysis are provided in theSupplementary Materials.

### 2.5 Statistical analysis of metrics

For the statistical analysis of the three metrics (positive mean strength, negative mean strength, and FCC), we employed a linear mixed effects model (LMEM) approach. Each model used one of the metrics as the dependent variable, with preprocessing pipeline, lesion size range, session, and their interactions as fixed effects, and subject ID and session as random effects. By modeling subject ID as a random effect with random slopes for session, we accounted for the dependent data arising from multiple measures for the same subject under multiple pipelines and sessions. Lesion size was transformed into a categorical variable and sizes were grouped into four equal groups (0–3cm^3^,3–14cm^3^,14–41cm^3^, 41–223cm^3^). In total, we fitted six models, one per metric and lesion type (hemorrhagic and ischemic). This approach allowed us to assess the influence of preprocessing pipelines while accounting for patient-specific differences and repeated measures across sessions.

After fitting the models, we conducted ANOVA to test the overall significance of the fixed effects in the model (such as lesion size, session, or preprocessing pipeline). This step helps to identify which factors and interactions contribute meaningfully to the observed variance in the data.

Following the ANOVA, we performed Tukey’s Honest Significant Difference (HSD) post hoc tests. Tukey’s HSD is used to make pairwise comparisons between the levels of the fixed effects while controlling for type I error in multiple comparisons. This test helps to pinpoint specific differences between preprocessing pipelines, lesion sizes, and sessions, providing a more detailed understanding of how each factor and their interactions affect the evaluation metrics.

LMEM analysis was conducted in R, using “lme4” package (D. Bates et al., 2015). For computing p-values of ANOVA tests, we used package “lmerTest” (Kuznetsova et al., 2017). Finally, pairwise analysis was conducted using package “emmeans” (Lenth, 2024).

### 2.6 Visual evaluation

To visually see the effect of each pipeline on connectivity and especially connectivity of the stroke lesion, we computed the seed to voxels connectivity, with the stroke lesion as seed. The average signal from the region of interest (ROI) mask was computed and the Pearson correlation of this signal with the rest of the brain was computed.

### 2.7 Lesion location

To assess whether the impact of preprocessing pipelines varied by lesion location, we conducted a voxel-based lesion-symptom mapping (VLSM) analysis (E. Bates et al., 2003). This analysis examined the interaction between lesion presence at each voxel and preprocessing pipeline effects on connectivity metrics. Full details are provided in theSupplementary Materials.

### 2.8 Behavioral predictions

To assess the impact of different preprocessing pipelines on the prediction of behavioral scores from functional connectivity matrices in stroke patients, we closely followed the methodology described bySiegel, Ramsey, et al. (2016)andSalvalaggio et al. (2020). Specifically, we investigated the ability of connectivity matrices derived from each preprocessing pipeline, at 2 weeks poststroke, to predict behavioral impairments using machine learning models.

**Modeling**The following details closely match the previous studies fromSiegel, Ramsey, et al. (2016)andSalvalaggio et al. (2020), in order to enable a fair comparison that highlights the effect of preprocessing measures. To predict behavioral scores from the connectivity matrices, we employed ridge regression models. For each preprocessing pipeline, the connectivity matrices served as input features, and the behavioral scores were the output variables. Principal Component Analysis (PCA) was first applied to reduce the dimensionality of the connectivity matrices while retaining 95% of the variance. Then, ridge regression models were trained on the reduced connectivity matrices to predict behavioral scores using a leave-one-(patient)-out cross validation loop (LOOCV). Finally, prediction accuracy was assessed using the coefficient of determination (R²) between the predicted and actual behavioral scores. The predictive performance of models derived from the connectivity matrices of each preprocessing pipeline was compared based on this R² value. Since we used LOOCV, each preprocessing pipeline produces only a single R² value, reflecting the overall model performance across all data. This presents a challenge in comparing pipelines, as we lack multiple R² values to assess variability or perform statistical comparisons. However, in this context, the single R² value remains informative, providing a relative measure of how well each preprocessing pipeline preserved connectivity information relevant to behavioral outcomes across the entire dataset.

## 3 Results

### 3.1 Quality control (QC) metrics

**Hemodynamic lags**Hemodynamic lags were calculated to assess temporal delays in rsfMRI imaging signals. Patients exhibiting a mean hemodynamic lag exceeding 1 s in the affected hemisphere were excluded from the analysis. This resulted in six patients at 2 weeks, two at 3 months, and four at 1 year post-stroke being excluded. For the remaining patients, the mean lags reported were0.47±0.16s at 2 weeks,0.43±0.18s at 3 months, and0.41±0.17s at 1 year. Representative examples of hemodynamic lag maps are given inSupplementary Figure S1for a patient with limited hemodynamic lag andSupplementary Figure S2for a patient with mean hemodynamic lag higher than 1 s.

**Movements**Mean FD was calculated to assess head motion. The mean FD across all sessions was approximately0.2mm, indicating minimal movement artifacts in the retained data.

**ICA lesion**The mean number of independent components overlapping with the lesion mask was assessed. On average, one ICA component overlapped with the lesion for all three sessions, with a mean number of components of approximately 0.1 for lesions ranging from0to3cm^3^,0.5for lesions from3to14cm^3^,1.5for lesions from14to41cm^3^, and2.8for lesions larger than41cm^3^.

### 3.2 Metrics

Results of the LMEM models are shown inFigure 3for ischemic stroke and hemorrhagic stroke, and post hoc tests are shown inFigures 4and5. Detailed results of the LMEM models are provided inSupplementary Tables S1 to S6.

**Fig. 3.**
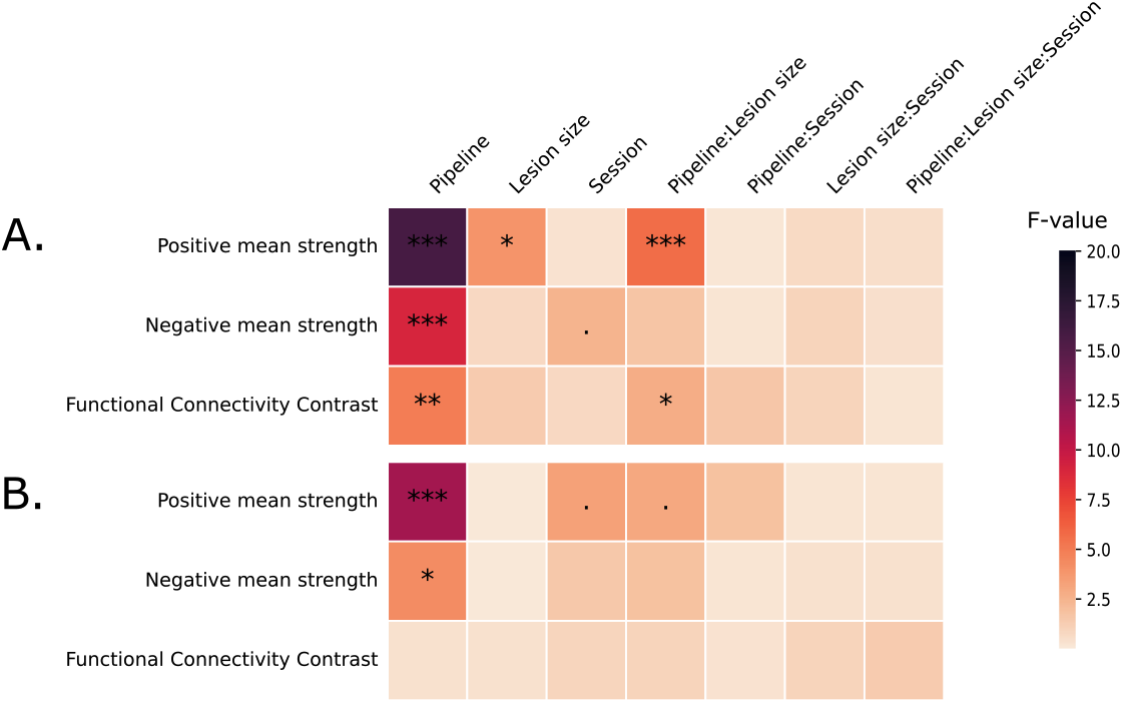
Results of LMEM and ANOVA for (A) ischemic stroke patients and (B) hemorrhagic stroke patients. “:” denotes interaction effects in LMEM. F value of the statistical test are represented with the color scale and the levels of statistical significance are denoted as:p≤0.1: “.”,p≤0.05: “*”,p≤0.01: “**”,p≤0.001: “***”,p≤0.0001: “****”. We can see that “pipeline” has a significant effect on most metrics for the two types of lesions.

**Fig. 4.**
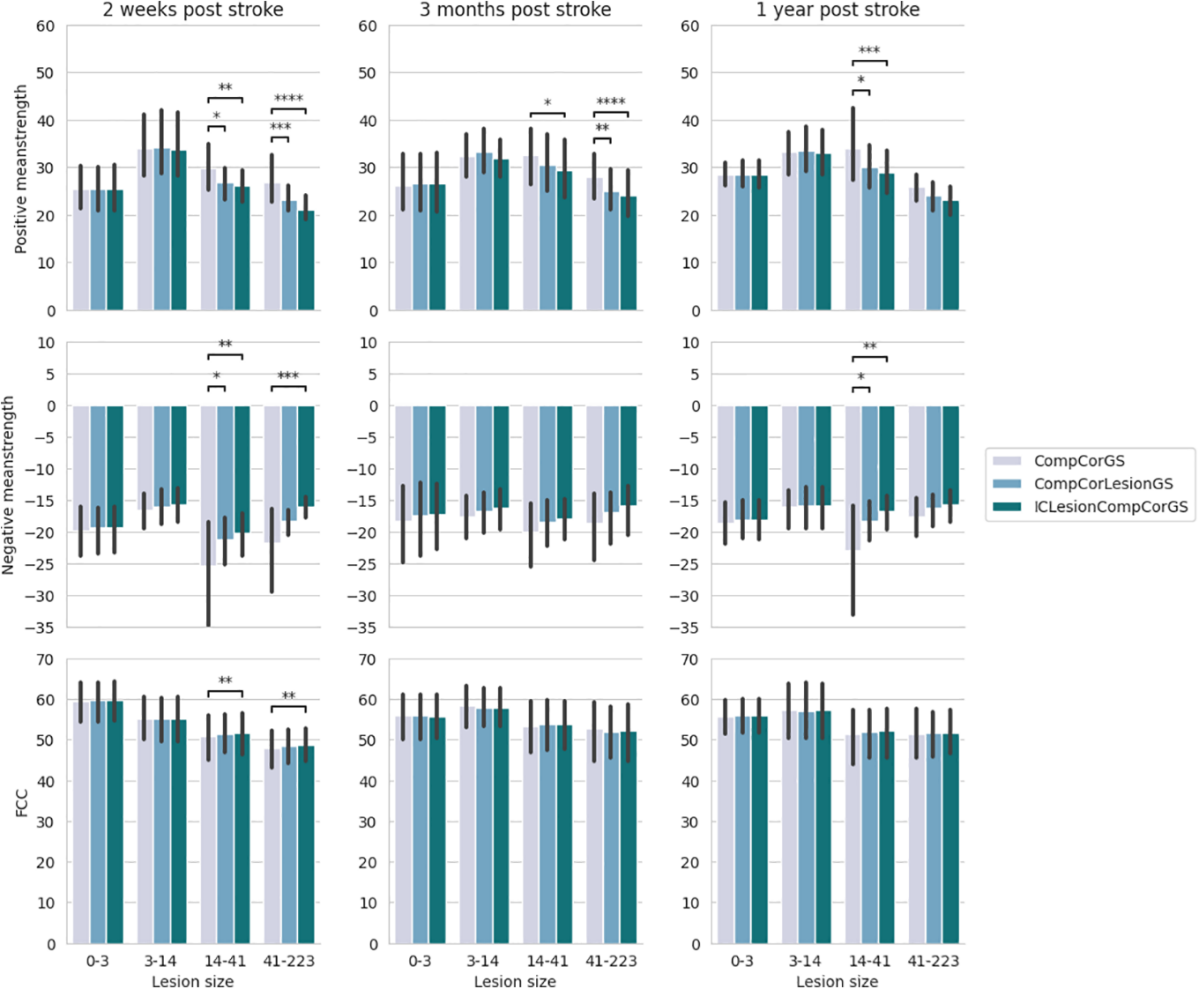
Results of the post hoc tests for ischemic stroke patients for the three metrics (positive mean strength, negative mean strength, and functional connectivity contrast), the three sessions (2 weeks, 3 months, and 1 year post stroke), and for different lesion sizes (in cm³); the bars represent the confidence intervals and the levels of statistical significance are denoted as:p≤0.1: “.”,p≤0.05: “*”,p≤0.01: “**”,p≤0.001: “***”,p≤0.0001: “****”.

**Mean strength**The LMEM results for positive mean strength in patients with ischemic strokes (Fig. 3A) revealed significant effects of the pipeline (p≤0.001), lesion size (p≤0.01), and the interaction between lesion size and pipeline (≤0.001). Post hoc tests (Fig. 4) provided further details, showing that the ICLesionCompCorGS pipeline exhibited smaller positive mean strength than the CompCorGS pipeline across all three sessions for larger lesions (>14 cm³). At 2 weeks post-stroke, the ICLesionCompCorGS pipeline demonstrated significantly smaller mean strength compared with the CompCorGS and CompCorLesionGS pipelines (p≤0.05andp≤0.01, respectively) for patients with lesion sizes between 14 cm³ and 41 cm³. This difference was even more pronounced in patients with lesions >41 cm³ (p≤0.001andp≤0.0001). It is important to note that we aim for the positive mean strength to be small, as this indicates minimal spurious connectivity between the lesion site and other brain regions. This is because regions overlapping with stroke lesions typically consist of non-functional tissue, which should not exhibit strong connectivity with other brain areas.

Three months post-stroke, this trend persisted, particularly for the largest lesions, with more pronounced differences between the ICLesionCompCorGS pipeline and the CompCorGS pipeline, although these differences were no longer significant 1 year post-stroke.

For patients with hemorrhagic strokes (Fig. 3B), although the results were less significant overall, a notable difference was still observed between pipelines, withp≤0.0001. As shown inFigure 5, the CompCorGS pipeline exhibited significantly greater mean strength than the ICLesionCompCorGS pipeline at 3 months and 1 year post-stroke (p≤0.0001andp≤0.001, respectively) for the largest lesion group.

**Fig. 5.**
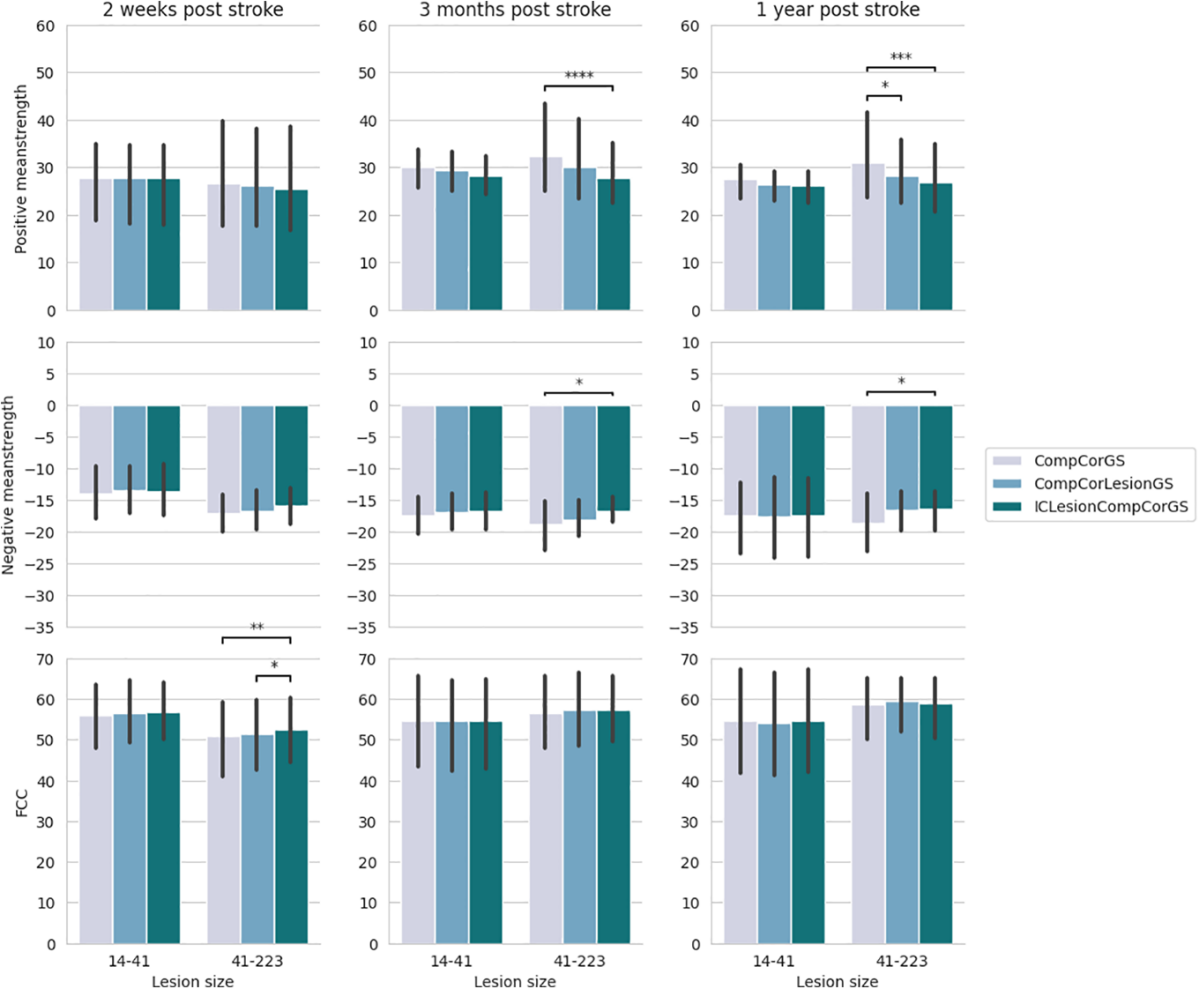
Results of the post-hoc tests for hemorrhagic stroke patients for the three metrics (positive mean strength, negative mean strength, and functional connectivity contrast), the three sessions (2 weeks, 3 months, and 1 year post-stroke), and for different lesion sizes (in cm³). The levels of statistical significance are denoted as:p≤0.1: “.”,p≤0.05: “*”,p≤0.01: “**”,p≤0.001: “***”,p≤0.0001: “****”.

It is worth noting that the results consistently followed the same trend CompCorGS greater than CompCorLesionGS greater than ICLesionCompCorGS—even when the differences were not statistically significant.

Negative mean strength, while more challenging to interpret, also showed significant effects of the preprocessing pipeline for both ischemic (p≤0.001) and hemorrhagic strokes (p≤0.05) (seeFig. 3). The CompCorGS pipeline produced stronger negative correlations than the ICLesionCompCorGS pipeline. Specifically, at 2 weeks post-stroke, significant differences were observed for patients with lesion sizes between 14 and 41 cm³ (size 3,p≤0.01) and for those with lesions larger than 41 cm³ (size 4,p≤0.001). At 3 months post-stroke, the CompCorGS pipeline continued to show stronger negative correlations for the size 3 group (p≤0.01). Similarly, for hemorrhagic strokes, significant differences were found at 3 months (p≤0.05) and at 1 year post-stroke (p≤0.05) (Supplementary Table S6).

**FCC**The results for FCC showed a significant effect of the preprocessing pipeline for ischemic stroke patients (p≤0.01) and an interaction between lesion size and pipeline (p≤0.01) (Fig. 3). Specifically, for patients with ischemic strokes (Fig. 4), the ICLesionCompCorGS pipeline demonstrated significantly higher FCC values compared with the CompCorGS pipeline during the 2-week post-stroke session for patients with lesions larger than 14 cm³. The differences were statistically significant for patients with lesion sizes ranging from 14 to 41 cm³ (p≤0.01) and for those with lesions greater than 41 cm³ (p≤0.01).

Similar trends were observed for patients with hemorrhagic strokes (Fig. 5), where the ICLesionCompCorGS pipeline outperformed the CompCorGS pipeline 2 weeks post-stroke (p≤0.01). However, the effect was less pronounced in sessions further from the stroke onset, with no significant differences observed.

Finally, effects of the pipeline on FCC were less pronounced in sessions further from the stroke onset, with no significant differences observed.

**Q value**We observed a slight reduction in Q-values for stroke-specific pipelines, particularly in patients with larger lesions and early post-stroke sessions. The full statistical results are provided in Supplementary Materials,Figures S4, S5, and S6.

Additionally, to evaluate the impact of global signal regression (GSR), we repeated all analyses without applying GSR. Results showed that the main tendencies remained consistent across mean strength and functional connectivity contrast (FCC). Negative mean strength behaved differently, which is expected given that GSR tends to introduce global negative correlations. These additional analyses are provided in the Supplementary Materials,Figures S7, S8, and S9.

### 3.3 Visual evaluation

**Fig. 6.**
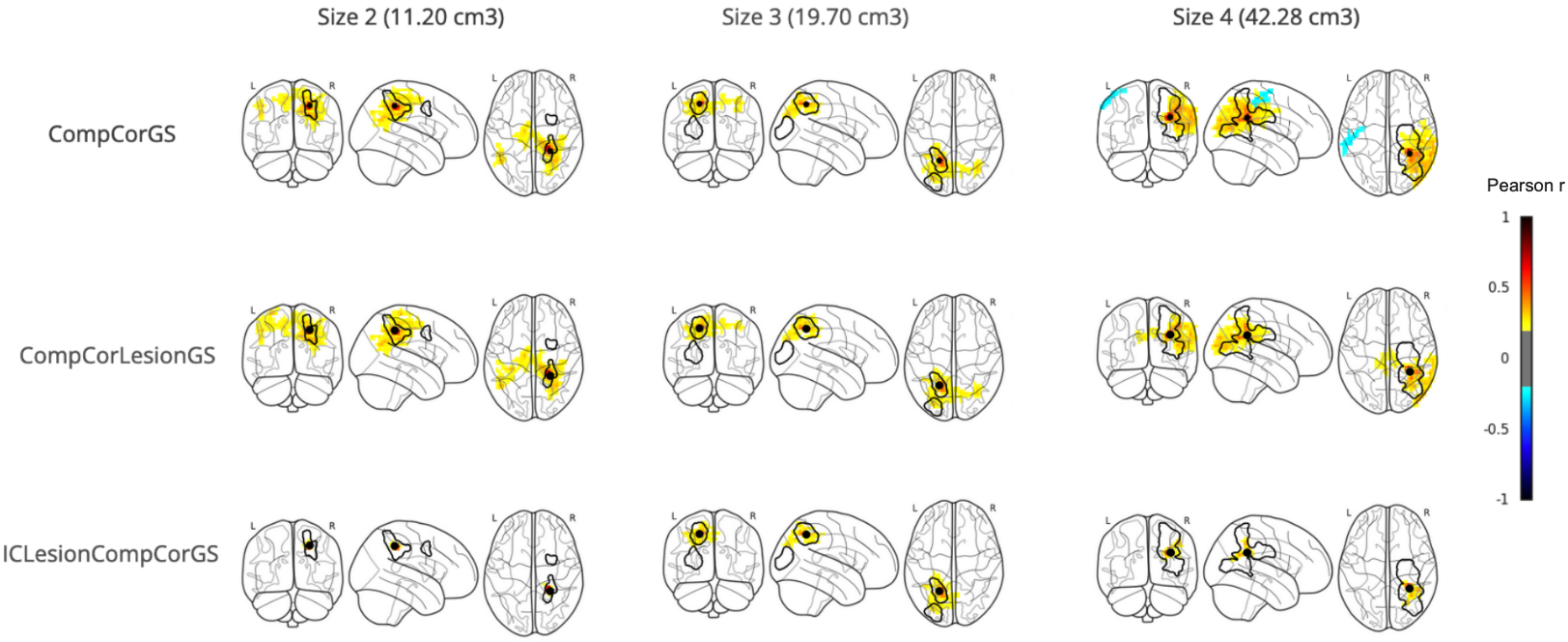
Seed-based connectivity maps for three patients representative of the three largest lesion size ranges. The first two rows display seed-based connectivity computed using the CompCorGS and CompCorLesionGS pipelines, respectively, showing connectivity extending into non-lesioned brain areas. The third row represents the ICLesionCompCorGS pipeline, demonstrating significantly reduced connectivity with the lesion.

**Lesion connectivity**To further illustrate the effect of different pipelines on connectivity,Figure 6shows seed-based connectivity using the lesions as the seed for representative patients from three of the four subgroups of lesion sizes at the acute phase (2 weeks post-stroke). For all three examples, the CompCorGS pipeline showed connectivity extending into non-lesion brain areas. The ICLesionCompCorGS pipeline, however, minimized these artifacts, highlighting the effectiveness of incorporating lesion-specific confounds and ICA-based components in preprocessing especially for patients with larger lesions.

### 3.4 Lesion location

We further explored whether lesion location influenced preprocessing effectiveness. The VLSM analysis revealed that preprocessing effects were most pronounced when lesions were located in specific brain regions, including visual, somatosensory, and motor-related pathways. Interestingly, in these regions, stroke-specific pipelines had a greater impact on reducing spurious connectivity and improving network differentiation. Full results are available in the Supplementary Materials,Figures S10, S11, and S12.

### 3.5 Behavioral predictions

Table 1summarizes the predictive performance of the different preprocessing pipelines on behavioral outcomes.

**Table 1.** Results of behavioral predictions.

	Subjects	CompCorGS	CompCorLesionGS	ICLesionCompCorGS
Language	100	R² = 0.17; ncomp = 83	R² = 0.22; ncomp = 85	R² = 0.20; ncomp = 86
Motor left (right lesion)	46	R² = 0.00†; ncomp = 40	R² = 0.00†; ncomp = 40	R² = 0.00†; ncomp = 40
Motor right (left lesion)	46	R² = 0.02†; ncomp = 39	R² = 0.02†; ncomp = 39	R² = 0.02†; ncomp = 39
Visual left (right lesion)	22	R² = 0.00†; ncomp = 19	R² = 0.00†; ncomp = 19	R² = 0.00†; ncomp = 19
Visual right (left lesion)	28	R² = 0.38; ncomp = 24	R² = 0.33; ncomp = 24	R² = 0.30; ncomp = 24
Verbal memory	79	R² = 0.13; ncomp = 68	R² = 0.13; ncomp = 68	R² = 0.13; ncomp = 68
Spatial memory	79	R² = 0.17; ncomp = 68	R² = 0.20; ncomp = 68	R² = 0.19; ncomp = 68
Attention (visual bias field)	87	R² = 0.26; ncomp = 75	R² = 0.20; ncomp = 75	R² = 0.24; ncomp = 75

ncom: Number of PCA components, †non-significant models.

Columns show results for the three preprocessing pipelines and the number of subjects included in the model. Each row corresponds to a different behavioral measure, detailing the R^2^value and the number of principal components used. Overall, the results indicate marginal differences between the pipelines. Notably, the motor function predictions for both left and right lesions are poor, with non-significant models marked by the †symbol.

The results of the behavioral predictions show small variations in the performance of different preprocessing pipelines. For language prediction, the CompCorLesionGS pipeline demonstrated the highest accuracy, with an R² value of 0.22, slightly surpassing ICLesionCompCorGS at 0.20 and CompCorGS at 0.17. Motor function predictions were generally poor across all pipelines, with R² values near zero, indicating non-significant models.

In terms of visual functions, the prediction accuracy varied based on the side of the lesion. For patients with right-sided lesions (affecting left visual function), the predictive performance was near zero for all pipelines. However, for left-sided lesions (affecting right visual function), the CompCorGS pipeline performed best with an R² of 0.38, followed by CompCorLesionGS at 0.33 and ICLesionCompCorGS at 0.30.

Memory function predictions showed mixed results. For verbal memory, all pipelines performed equally with an R² of 0.13. However, for spatial memory, CompCorLesionGS exhibited slightly better performance (R² = 0.20) than CompCorGS (R² = 0.17) and ICLesionCompCorGS (R² = 0.19). For attention, the CompCorGS pipeline yielded the highest predictive accuracy (R² = 0.26), followed by ICLesionCompCorGS (R² = 0.24) and CompCorLesionGS (R² = 0.20).

Overall, the inclusion of lesion-specific and ICA-based components in the ICLesionCompCorGS pipeline has no clear effect on predictive performance, although it slightly enhances the results in certain domains, particularly for language and spatial memory. However, considering the observed differences and the fact that each pipeline produces only a single R² value, we cannot conclude that there are substantial differences in predictive performance across the pipelines.

## 4 Discussion

In this paper, we addressed preprocessing challenges in fMRI data analysis for stroke patients, whose neurovascular disruptions introduce artifacts that complicate the interpretation of functional connectivity. Currently, there is no consensus on the best preprocessing approach for stroke fMRI data. Recognizing this gap, this study provided a comparative analysis of specifically designed preprocessing pipelines to effectively counter these stroke-related artifacts. By systematically assessing various strategies, we enhanced the reliability and interpretability of connectivity findings. Moreover, by releasing the pipelines as an open-source toolkit (fMRIStroke) compatible with fMRIprep, we ensured the replicability of our methods and promoted broader validation and adoption within the research community.

Among the tested pipelines, the ICLesionCompCorGS emerged as the most effective. It significantly reduced artifacts, particularly from lesioned areas, and minimized spurious correlations, thereby providing clearer and more reliable functional connectivity maps. This study’s findings underscore the importance of tailored preprocessing strategies in neuroimaging research for stroke.

### 4.1 Quality control metrics

Effective QC measures are essential for ensuring the accuracy of functional connectivity analysis (Parkes et al., 2018), especially considering the neurovascular complications inherent to stroke, as emphasized in studies such as those bySiegel et al. (2017).

**Addressing motion**One of the challenges in preprocessing fMRI data, particularly in stroke patients, is managing head movement artifacts that can introduce spurious connectivity measures. In this study, we excluded high-motion subjects to ensure that the retained data were of sufficient quality. Additionally, to mitigate any remaining residual movement artifacts, we included motion estimates as confounding regressors in the denoising process across all preprocessing pipelines. This approach helps to account for the variance introduced by movement and reduces its impact on the final connectivity measures.

**Addressing hemodynamic lag in stroke fMRI**Following common methods to estimate hemodynamic lags from resting-state fMRI (Siegel, Snyder, et al., 2016), we found that these lags were prevalent in stroke patients and were not homogeneous across the brain, often extending beyond stroke lesions. At 2 weeks post-stroke, a larger proportion of patients exhibited these lags, consistent with previous results (Siegel, Snyder, et al., 2016), which reported that over30%of patients showed significant lags at this stage, decreasing to around10%by 1 year. As highlighted bySiegel et al. (2017)andChristen et al. (2015), hemodynamic lags can distort connectivity maps, often causing underestimations of connectivity (false negatives) or misinterpretations of the brain’s functional integration. For instance,Siegel et al. (2017)noted that many studies showing homotopic anti-correlations in stroke patients might be significantly impacted by these lags. Various methods have been proposed to correct for these hemodynamic lags (Christen et al., 2015;Frederick et al., 2012;Siegel, Snyder, et al., 2016), such as shifting time series by their computed lag. However, these correction techniques have notable disadvantages. While they might improve the accuracy of FC maps, they do not address the underlying issue of altered BOLD signal frequency content and might introduce new artifacts. Additionally, shifting time series could interfere with other noise correction techniques. Given these drawbacks, we followedSiegel et al. (2017)’s recommendation to exclude patients with extensive lags. Specifically, we excluded those with a mean lag exceeding 1 s in the affected hemisphere. After this exclusion, the remaining patients had a mean lag below0.5s, aligning with the guidelines and enhancing the reliability of our following measurements.

**ICA effectively identifies lesion-driven artifacts**In this study, we implemented the same ICA-based approach as described byYourganov et al. (2017)to address the challenges posed by stroke lesions in resting-state functional connectivity (rs-FC) analyses.Yourganov et al. (2017)demonstrated the effectiveness of ICA in isolating and removing lesion-driven artifacts, showing that their ICA-based pipeline detected at least one lesion-driven independent component in the majority of participants (54 out of 75), with a clear correlation between lesion size and the number of identified components. In this study, we successfully replicated the findings ofYourganov et al. (2017). We identified ICA components overlapping with the lesion mask in a comparable proportion of patients (72 out of 106), with an average of about one significant component per patient. Importantly, these components often extended beyond the lesion itself, demonstrating ICA’s robust capability to isolate and detect lesion-driven artifacts.

### 4.2 Stroke-specific pipelines outperform standard pipeline across all metrics

Building upon the findings fromYourganov et al. (2017), we replicated their results showing that the ICA-based pipeline (ICLesionCompCorGS) effectively reduces artificially inflated functional connectivity (FC) values within lesioned areas. We expanded this analysis by not only focusing on the connectivity within the lesion mask but also examining how these regions connect to other parts of the brain by computing the mean strength of these connections. This approach showed that regions within the lesion mask could maintain spurious connections with distant brain areas.

Additionally, we introduced the FCC metric (Kassinopoulos & Mitsis, 2022) to provide a broader perspective on the impact of lesion-driven artifacts. By reflecting the extent to which expected functional differentiation is maintained, FCC serves as an indicator of network segregation and provides a measure of meaningful signal preservation. The FCC metric enabled us to assess the overall functional network organization of the brain with fewer assumptions about the lesion site. This approach aligns with methodologies from prior studies evaluating preprocessing pipelines focusing on healthy subjects such asParkes et al. (2018)andShirer et al. (2015). Our results demonstrated that the ICA method resulted in higher FCC values, indicating that the impact of lesion-driven artifacts extends beyond the immediate lesion site and affects broader network integrity. The increase or stability in FCC further suggests that adding lesion-related regressors effectively reduces spurious connectivity without removing true functional signal.

Our findings also highlighted significant variations in the effectiveness of preprocessing pipelines based on lesion size and session timing. Consistent with the findings ofYourganov et al. (2017), we observed that the influence of the pipeline is more pronounced with larger lesions, where the ICA-based corrections were particularly effective in reducing spurious correlations caused by lesion artifacts. Additionally, we extended their results by including a wider range of stroke recovery stages, assessing patients from as early as 2 weeks post-stroke through to later recovery phases and found that the effectiveness of the pipeline is also dependent on the session, having a higher impact for sessions closest to the stroke onset. Interestingly, for hemorrhagic strokes, although the overall trends were similar, the improvements were less pronounced compared with ischemic strokes, particularly in later recovery stages.

To further explore preprocessing effects, we also examined network modularity using the Q-value metric (Ciric et al., 2017). Stroke-specific pipelines led to a small reduction in Q-values, particularly in larger lesions and early post-stroke sessions. However, it remains unclear whether this decrease reflects the removal of true functional signal or whether lesion-driven artifacts artificially inflated modularity estimates, making their removal appear as a reduction in modularity. Given this uncertainty, Q-value should be interpreted with caution. Importantly, since FCC remained stable or improved, functional connectivity within predefined networks appears to have been preserved or even improved.

These findings highlight the significant impact of preprocessing strategies, which vary according to the distinct characteristics of the stroke and the timing of the imaging session. Our results suggest that ICLesionCompCorGS may be a preferable regressor combination for reducing lesion-driven artifacts while preserving functional network structure especially when dealing with large lesions and data acquired close to lesion onset. Additionally, given the influence of hemodynamic lag on functional connectivity estimates, excluding patients with a mean lag in the affected hemisphere exceeding 1 s could help improve the reliability of connectivity analyses.

### 4.3 Behavioral predictions show marginal differences

To further evaluate the impact of preprocessing pipelines on functional connectivity, we conducted a study of behavioral predictions. We followed the approach taken byYourganov et al. (2017), which utilized behavioral prediction as a means to compare the effectiveness of various preprocessing pipelines in handling fMRI data from stroke patients.Yourganov et al.’s (2017)research highlighted that integrating adjustments for lesion-driven artifacts significantly enhanced the predictive accuracy of behavioral outcomes. Inspired by this approach, we adopted a similar comparative methodology. Specifically we focused on models previously explored bySiegel, Ramsey, et al. (2016)andSalvalaggio et al. (2020)using the same dataset. This methodological choice underlines our focus on comparing pipeline performance, without attempting to fine-tune the prediction models themselves. Contrary toYourganov et al. (2017), our study revealed only marginal differences in the different pipelines predicting performances. Predictions for motor deficits were particularly poor across all pipelines, aligning with previous findings bySiegel et al. (2017)andSalvalaggio et al. (2020), which suggested that lesion locations typically provide more reliable predictions than functional connectivity for these deficits. While the CompCorLesionGS and ICLesionCompCorGS pipelines demonstrated a slight advantage in predicting language functions, no single approach consistently outperformed others across most cognitive domains.

### 4.4 Limitation and perspectives

This study demonstrated that tailored preprocessing pipelines significantly enhance the reliability of functional connectivity measures in stroke patients. To build on this foundation, future research could explore several directions. Firstly, the sample size, while adequate for initial insights, is relatively small, particularly considering the variability in stroke lesions and patient demographics. The representation of hemorrhagic stroke cases for instance is relatively low, limiting robust conclusions for this subgroup. Larger studies with a more balanced representation of stroke types would help achieve more robust and generalizable findings, although larger stroke datasets are difficult to obtain.

Secondly, the decision not to correct for hemodynamic lags, while avoiding potential new artifacts, may limit the ability to fully address connectivity distortions caused by these lags and results in excluding patients from analysis, further research could be conducted for more sophisticated lag correction methods.

Additionally, our study focused on specific metrics to evaluate preprocessing pipelines. Incorporating new metrics that capture additional dimensions of functional connectivity and network dynamics could also provide more comprehensive assessment of the effect of preprocessing pipelines.

Moreover, ICA-based lesion artifact removal requires sufficient scan length for stable decomposition. While we excluded patients with excessively short scans, ICA-based regressors may be less reliable in datasets with shorter acquisitions, potentially making ICLesionCompCorGS less optimal in such cases.

Our results also suggest that beyond lesion size, preprocessing effectiveness varies by lesion location. A voxel-based lesion-symptom mapping (VLSM) analysis revealed that certain regions, particularly in visual, somatosensory, and motor-related pathways, were more affected by preprocessing choices. In these regions, stroke-specific pipelines had a greater impact on reducing spurious connectivity and improving network differentiation. These findings provide primary evidence that lesion location influences functional connectivity estimates. However, the reasons why these particular regions are more affected remain unclear. Future research could further investigate this effect and its implications for preprocessing strategies in stroke fMRI.

In terms of behavioral predictions, this study focused primarily on comparing preprocessing pipelines, which is why we replicated methodologies from previous studies. Future research could explore more sophisticated models to determine whether preprocessing pipelines have a greater impact and to potentially achieve better prediction results.

Finally, continued development and sharing of open-source tools such as fMRIStroke will facilitate broader validation and improvement of preprocessing pipelines, accelerating advancements and ensuring that these tools are robust, reliable, and widely applicable. By addressing these limitations and pursuing these future directions, the field can advance toward more reliable analyses of fMRI data in stroke patients, ultimately improving our understanding of stroke recovery.

## 5 Conclusion

In conclusion, our study systematically evaluated the effectiveness of different preprocessing pipelines for resting-state fMRI data in stroke patients. By comparing the traditional CompCorGS pipeline with stroke-specific adaptations, including CompCorLesionGS and ICLesionCompCorGS, we aimed to enhance the reliability and interpretability of functional connectivity measures. Our findings indicate that the ICLesionCompCorGS pipeline significantly reduces artifacts, particularly from lesioned areas, and minimizes spurious correlations, thereby providing clearer and more reliable functional connectivity maps. Quality control measures, particularly addressing hemodynamic lags and motion artifacts, were crucial in ensuring the accuracy of our analyses. Despite the observed improvements, our study also highlighted the limitations and challenges inherent in preprocessing fMRI data from stroke patients, such as sample size variability and the presence of hemodynamic lags.

Future research should focus on addressing these limitations, standardizing preprocessing protocols, and exploring new methods to correct for stroke-related artifacts without introducing additional biases. By advancing these areas, we can improve the robustness of stroke fMRI analyses and contribute to a better understanding of brain network reorganization and recovery mechanisms in patients suffering from cardiovascular diseases.

## Supplementary Material

Supplementary Material

## Data Availability

The dataset utilized in this study is available as presented inCorbetta et al. (2015). The preprocessing code developed and used for the study, including the custom pipeline (fMRIStroke), is openly accessible athttps://github.com/alixlam/fmristroke.
