# Supplementary material for: Evaluating lesion-specific preprocessing pipelines for rs-fMRI in stroke patients: Impact on functional connectivity and behavioral prediction
Source: Imaging Neurosci (Camb). 2025 May 30;3:IMAG.a.6. doi: 10.1162/IMAG.a.6 (PMC12320005; doi:10.1162/IMAG.a.6)
Supplement: Supplementary Material [file imag.a.6_supp.pdf]

1 Methods

1.1 Q value as an additional metric

We computed the Q-value, a modularity metric presented by [Ciric et al., 2017], which quantifies the degree to which functional networks self-organize into distinct modules without assuming predefined structures. Unlike FCC, which measures functional connectivity contrast within established networks, the Q-value provides a data-driven assessment of network integrity. A decrease in Q-value may indicate either a loss of functional signal or the successful removal of artifact-driven spurious connections. To assess the impact of preprocessing on modularity, we applied a linear mixed-effects model (LMEM) to compare Q-values across pipelines while accounting for lesion size and session.

1.2 Pipelines without Global Signal Regression (GSR)

GSR remains a debated preprocessing step as some studies [Li et al., 2019] have argued that the global signal carries meaningful neural information and removing it may distort functional connectivity patterns and introduce spurious negative correlations. To address this concern and provide a more complete evaluation of preprocessing choices, we have conducted an additional analysis where GSR was removed from all tested pipelines (CompCor, CompCorLesion, ICLesionCompCor). We then repeated the same evaluation using our established metrics—Mean Strength, Negative Mean Strength, and Functional Connectivity Contrast (FCC)—applying Linear Mixed Effects Models (LMEM) and post-hoc tests.

1.3 Voxel-based Lesion-symptom Mapping : Analysis of Preprocessing Effects

To investigate the impact of lesion location on preprocessing pipeline performance, we conducted a voxel-based lesion-symptom mapping (VLSM) Bates et al. [2003] analysis on the two weeks session. The goal was to determine whether certain lesion locations were more affected by the choice of preprocessing pipeline. For each voxel in lesion space, we used our three connectivity measures—Positive Mean Strength, Negative Mean Strength, and Functional Connectivity Contrast (FCC)—as behavioral variables. We then examined whether these measures could be explained by lesion location (whether the voxel was affected by a lesion), preprocessing pipeline, and the interaction between lesion location and pipeline. The key factor of interest was the interaction term, as a significant interaction would indicate that the preprocessing pipeline had a differential impact depending on lesion location. At each voxel, we fitted a linear mixed-effects model of the form:

$$\text{Measure} \sim \text{Lesion} + \text{Pipeline} + \text{Lesion} \times \text{Pipeline} + (1 \mid \text{Subject})$$

where Measure represents one of the three connectivity metrics (Positive Mean Strength, Negative Mean Strength, FCC), Lesion is a binary variable indicating whether the voxel is affected by a lesion, Pipeline represents the preprocessing pipeline used, and Lesion × Pipeline is the interaction term assessing whether the impact of preprocessing differs depending on lesion location. Since each subject was tested on all preprocessing pipelines, we included a random intercept for Subject to account for repeated measures. The significance of the interaction term was assessed using an F-test, identifying regions where preprocessing had the strongest differential effect. For regions where the interaction term was significant, we further conducted paired t-tests between pipelines to determine the direction of the effect (i.e., whether stroke-specific pipelines increased or decreased the given connectivity measure). To control for multiple comparisons, we applied a Bonferroni correction with a threshold of $p \leq 0.01$ across all tested voxels. Only results surviving this correction were considered significant.

2 Supplementary results

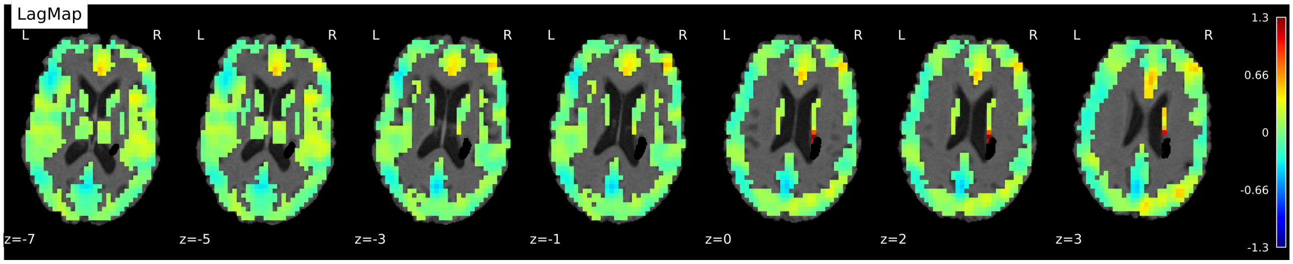

Figure S1: Example of patient with minimal hemodynamic lag, lesion mask is represented in black.

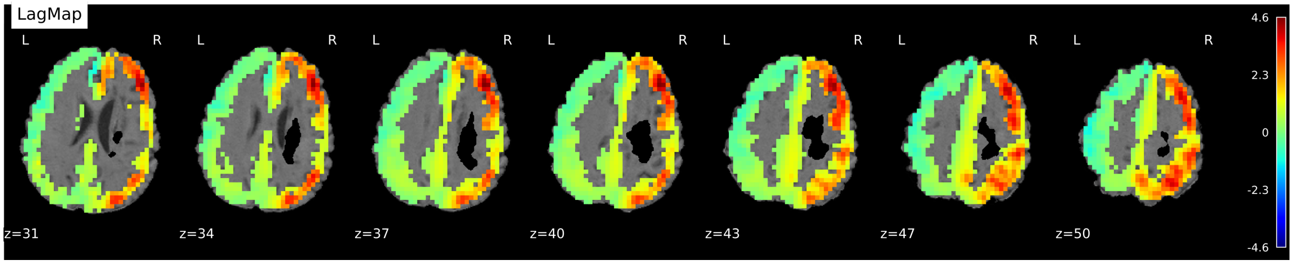

Figure S2: Example of patient with mean hemodynamic lag higher than one second, lesion mask is represented in black.

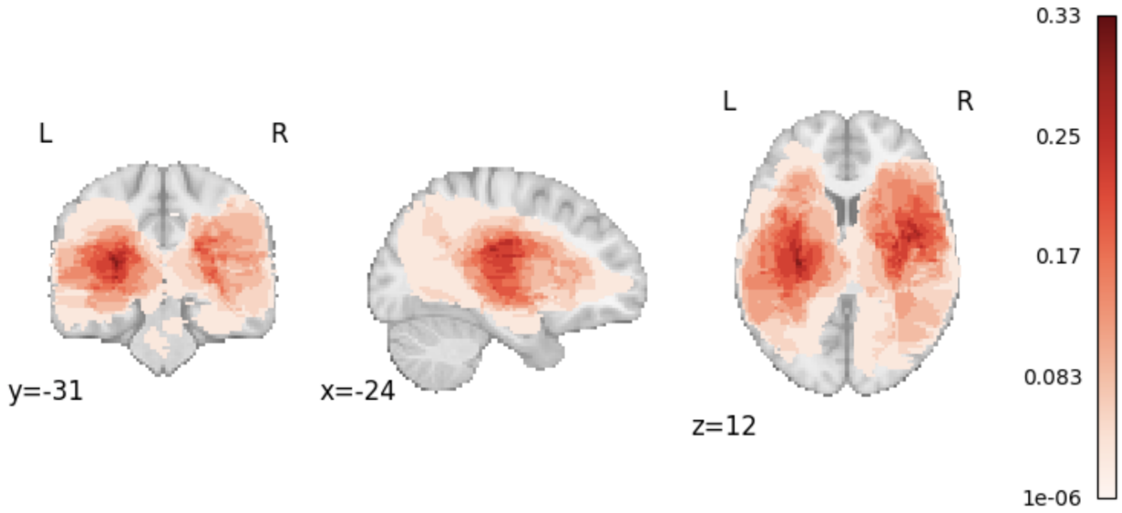

Figure S3: Lesion overlap of the evaluated patients.

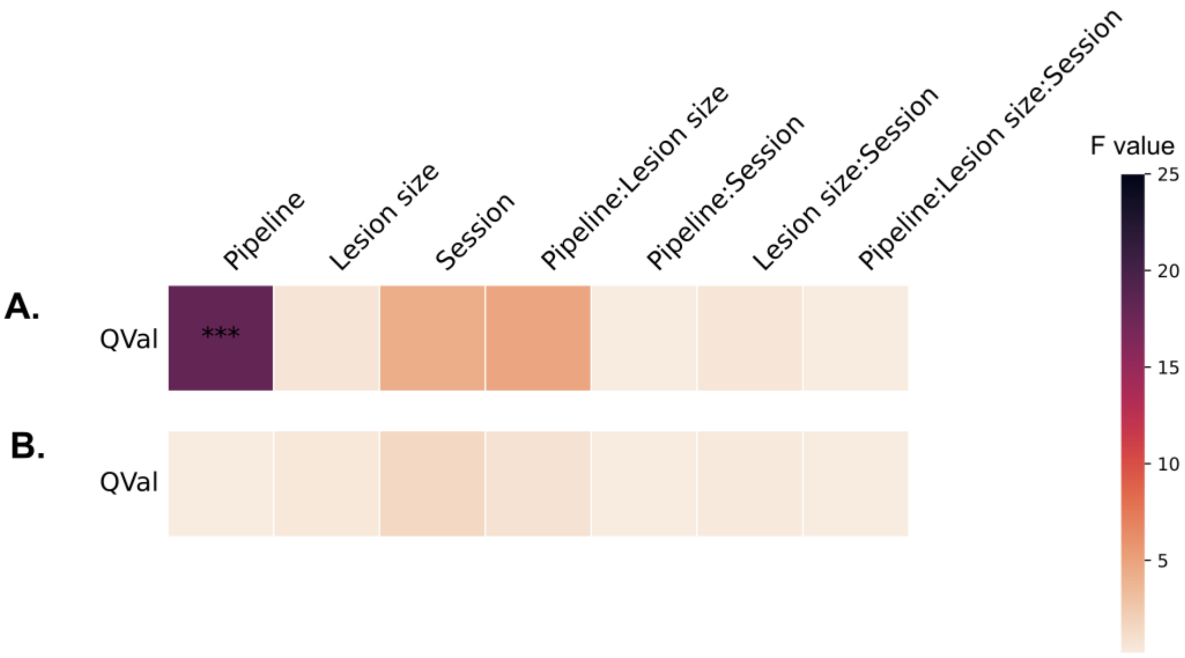

Figure S4: Results of LMEM and ANOVA for A. ischemic stroke patients and B. hemorrhagic stroke patients. ":" denote interaction effects in LMEM. F value of the statistical test are represented with the color scale and the levels of statistical significance are denoted as: $pleq 0.1$: ".", $p \leq 0.05$: "*", $p \leq 0.01$: "**", $p \leq 0.001$: "***", $p \leq 0.0001$: "****".

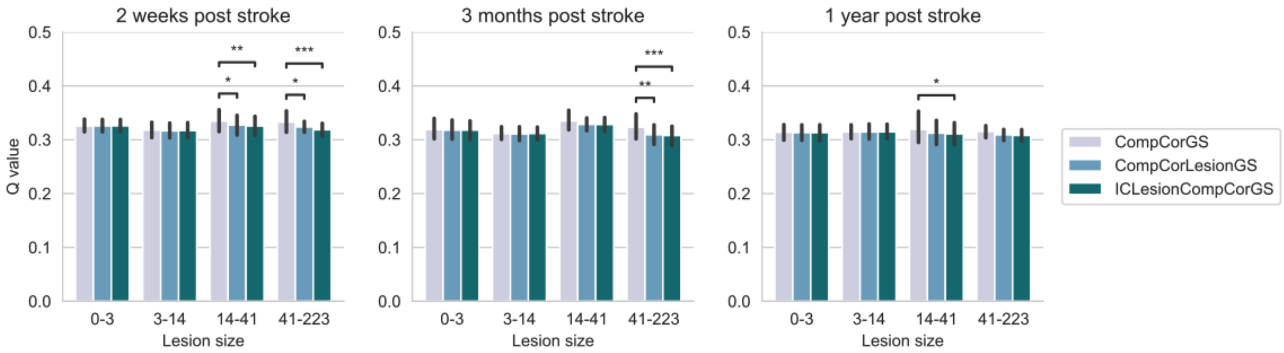

Figure S5: Results of the post-hoc tests for ischemic stroke patients for the Qvalue and the three sessions (2 weeks, 3 months and 1year post stroke);The levels of statistical significance are denoted as: $pleq 0.1$: ".", $p \leq 0.05$: "*", $p \leq 0.01$: "**", $p \leq 0.001$: "***", $p \leq 0.0001$: "****".

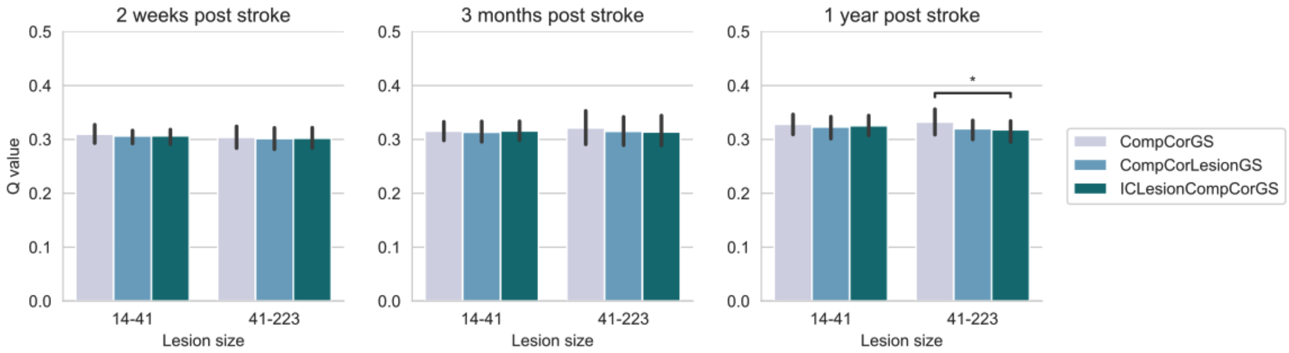

Figure S6: Results of the post-hoc tests for hemorrhagic stroke patients for the Qvalue and the three sessions (2 weeks, 3 months and 1year post stroke);The levels of statistical significance are denoted as: $pleq0.1$: ".", $p \leq 0.05$: "*", $p \leq 0.01$: "**", $p \leq 0.001$: "***", $p \leq 0.0001$: "****".

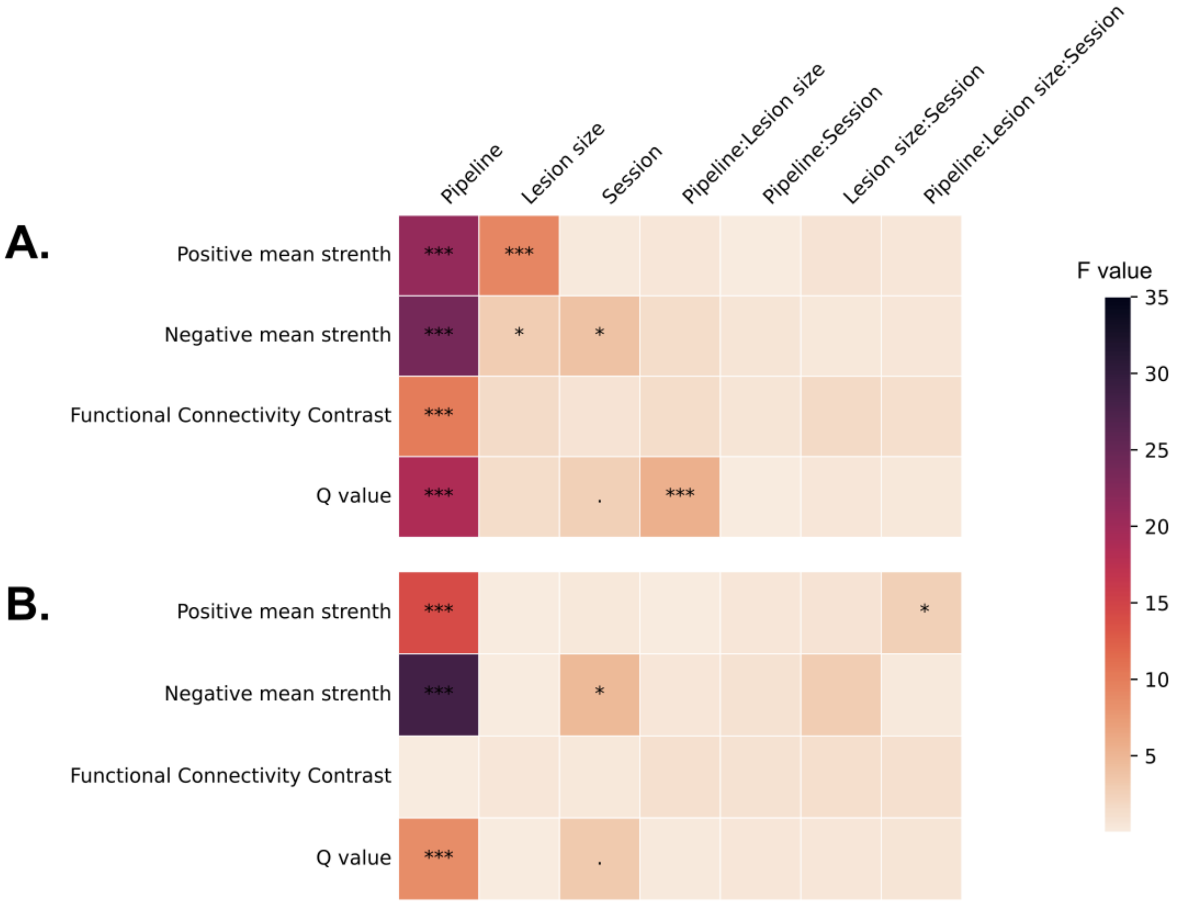

Figure S7: Results of LMEM and ANOVA for pipelines without GSR (CompCor, CompCorLesion, ICLesionCompCor) for A. ischemic stroke patients and B. hemorrhagic stroke patients. ":" denote interaction effects in LMEM. F value of the statistical test are represented with the color scale and the levels of statistical significance are denoted as: $pleq0.1$: ".", $p \leq 0.05$: "*", $p \leq 0.01$: "**", $p \leq 0.001$: "***", $p \leq 0.0001$: "****". We can see that "pipeline" has a significant effect on most metrics for the two types of lesions, here pipeline are CompCor, CompCorLesion, ICLesionCompCor.

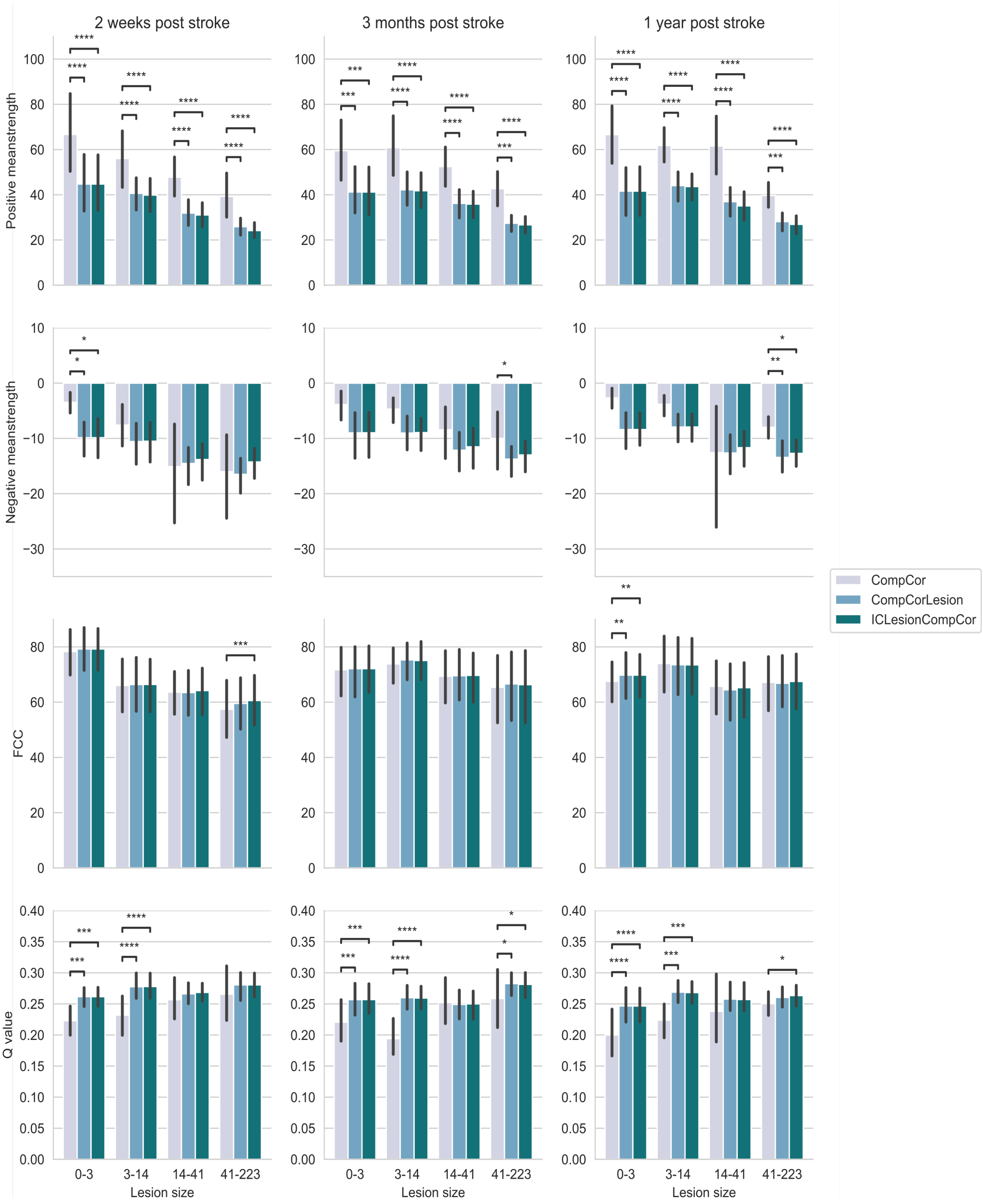

Figure S8: Results of the post-hoc tests for ischemic stroke patients for the four metrics (Positive mean strength, negative mean strength, Functional Connectivity Contrast and Qvalue) and the three sessions (2 weeks, 3 months and 1year post stroke); the bars represent the confidence intervals and the levels of statistical significance are denoted as: $pleq0.1$: ".", $p \leq 0.05$: "*", $p \leq 0.01$: "**", $p \leq 0.001$: "***", $p \leq 0.0001$: "****".

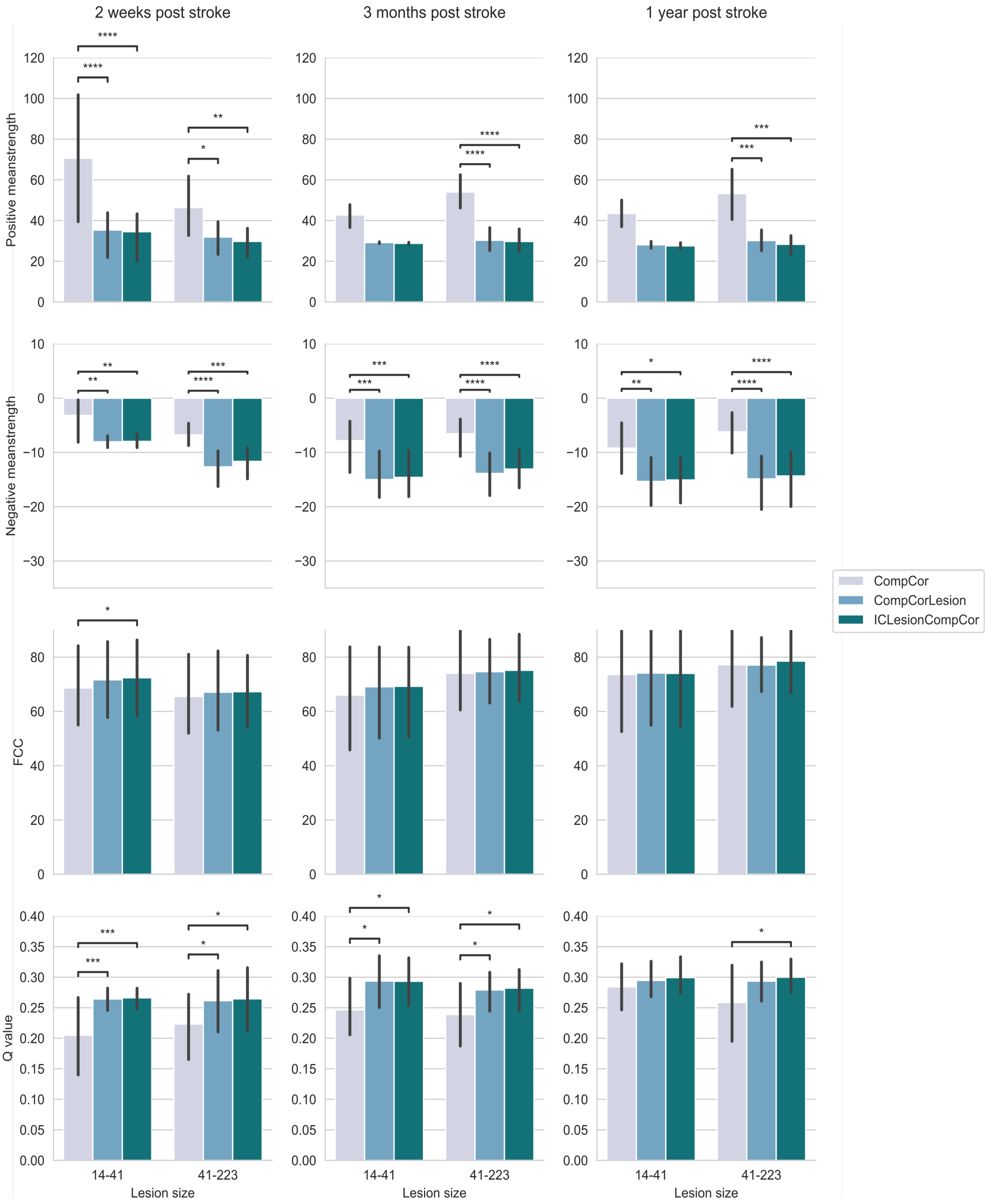

Figure S9: Results of the post-hoc tests for hemorrhagic stroke patients for the four metrics (Positive mean strength, negative mean strength, Functional Connectivity Contrast and Qvalue) and the three sessions (2 weeks, 3 months and 1year post stroke); the bars represent the confidence intervals and the levels of statistical significance are denoted as: $pleq0.1$: ".", $p \leq 0.05$: "*", $p \leq 0.01$: "**", $p \leq 0.001$: "***", $p \leq 0.0001$: "****".

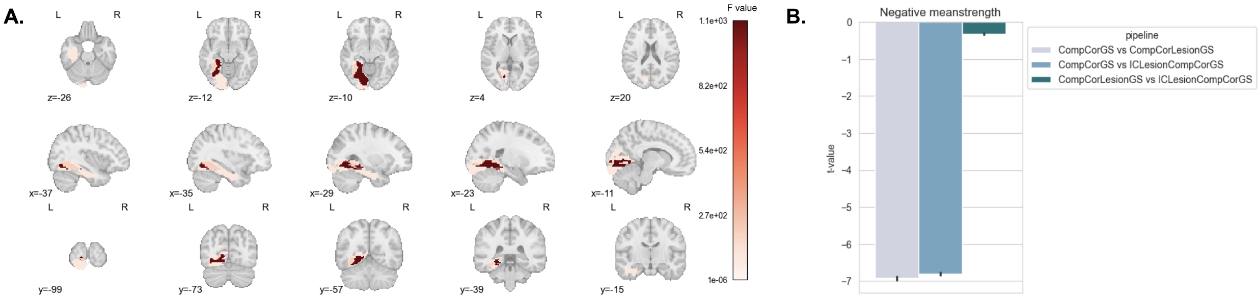

Figure S10: VLSM results showing the impact of lesion location on preprocessing pipeline performance for the Negative Mean Strength measure. A. Thresholded F-map highlighting voxels where the interaction between lesion presence and pipeline choice has a significant effect, after Bonferroni correction for multiple comparisons. These regions indicate where preprocessing pipelines significantly influence mean strength. B. Bar plot of t-values from paired t-tests comparing pipelines, illustrating the direction and magnitude of preprocessing effects. Results suggest that when lesions are located in the regions identified in panel A, stroke-specific preprocessing pipelines have a significantly greater effect in reducing Negative Mean Strength.

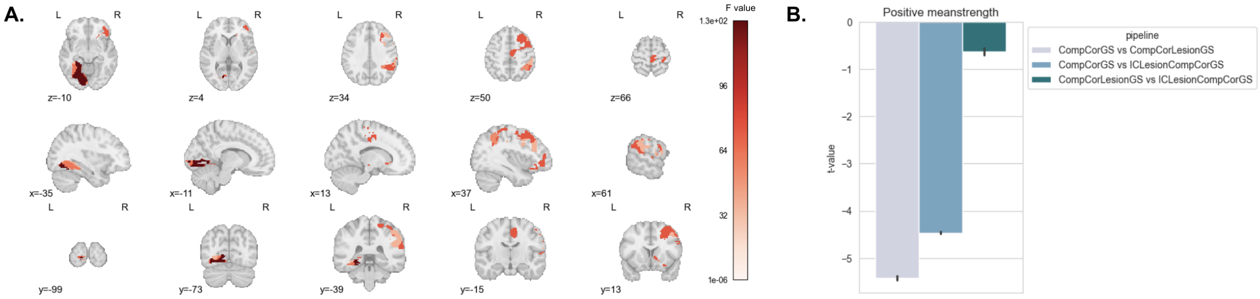

Figure S11: VLSM results showing the impact of lesion location on preprocessing pipeline performance for the Positive Mean Strength measure. A. Thresholded F-map highlighting voxels where the interaction between lesion presence and pipeline choice has a significant effect, after Bonferroni correction for multiple comparisons. These regions indicate where preprocessing pipelines significantly influence negative mean strength. B. Bar plot of t-values from paired t-tests comparing pipelines, illustrating the direction and magnitude of preprocessing effects. Results suggest that when lesions are located in the regions identified in panel A, stroke-specific preprocessing pipelines have a significantly greater effect in reducing Positive Mean Strength.

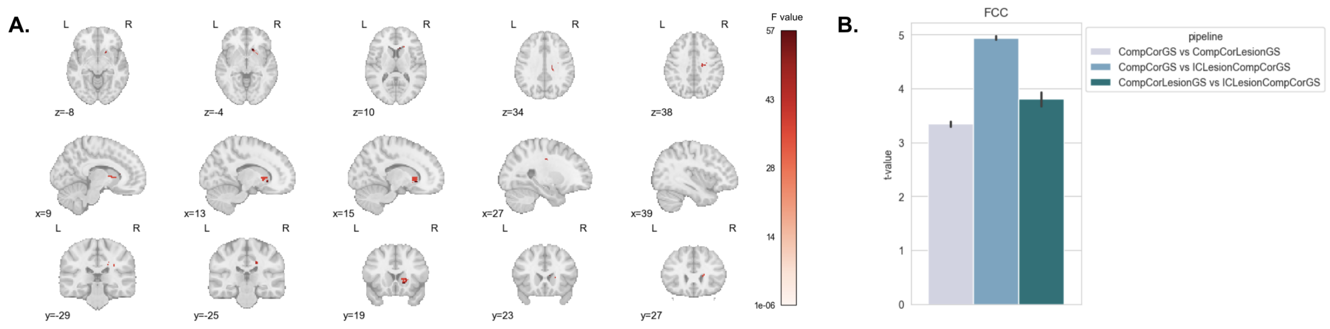

Figure S12: VLSM results showing the impact of lesion location on preprocessing pipeline performance for the Positive Mean Strength measure. A. Thresholded F-map highlighting voxels where the interaction between lesion presence and pipeline choice has a significant effect, after Bonferroni correction for multiple comparisons. These regions indicate where preprocessing pipelines significantly influence FCC. B. Bar plot of t-values from paired t-tests comparing pipelines, illustrating the direction and magnitude of preprocessing effects. Results suggest that when lesions are located in the regions identified in panel A, stroke-specific preprocessing pipelines have a significantly greater effect in increasing FCC .

2.1 Tables

ANOVA	F value	p-value		LMEM	Estimate	std Error
				Intercept	25.5534	4.1049
Pipeline	15.8546	2.807e-7	***	CompCorLesionGS	-0.1625	1.7390
				ICLesionCompCor	-0.1625	1.7390
Lesion Size	3.9886	0.012	*	3-14	8.1733	4.8934
				14-41	3.8625	4.8934
				41-223	3.1589	4.8934
Session	0.2845	0.753		3 months	0.0226	3.5539
				1 year	3.6930	4.1428
Pipeline:lesion size	5.6876	1.273e-5	***	CompCorLesionGS:3-14	0.6213	2.0731
				ICLesionCompCorGS:3-14	0.0583	2.0731
				CompCorLesionGS:14-41	-2.8171	2.0731
				ICLesionCompCorGS:14-41	-3.5135	2.0731
				CompCorLesionGS:41-225	-4.0999	2.0731
				ICLesionCompCorGS:41-225	-6.6867	2.0731
Pipeline:session	0.1721	0.953		CompCorLesionGS:3 months	0.5058	2.5457
				ICLesionCompCorGS:3 months	0.4739	2.5457
				CompCorLesionGS:1 year	0.0015	2.6564
				ICLesionCompCorGS:1 year	0.0015	2.6564
lesion size:session	0.6839	0.663		3-14:3 months	0.6889	4.3251
				14-41:3 months	2.3102	4.2566
				41-225:3 months	-0.8670	4.3187
				3-14:1 year	-3.7280	4.9514
				14-41:1 year	-0.5122	4.9471
				41-225:1 year	-3.7281	4.9514
pipeline:lesion size:session	0.5569	0.876		CompCorLesionGS:3-14:3 months	-0.1943	3.0793
				ICLesionCompCorGS:3-14:3 months	-1.0169	3.0793
				CompCorLesionGS:14-41:3 months	0.5334	3.0440
				ICLesionCompCorGS:14-41:3 months	0.1070	3.0440
				CompCorLesionGS:41-225:3 months	-0.0708	3.0605
				ICLesionCompCorGS:41-225:3 months	0.9614	3.0605
				CompCorLesionGS:3-14:1 year	-0.0721	3.1714
				ICLesionCompCorGS:3-14:1 year	-0.1377	3.1714
				CompCorLesionGS:14-41:1 year	-0.6831	3.1714
				ICLesionCompCorGS:14-41:1 year	-1.1271	3.1714
				CompCorLesionGS:41-225:1 year	2.8121	3.1714
				ICLesionCompCorGS:41-225:1year	4.0607	3.1714

Table S1: Results of ANOVA and LMEM of ischemic stroke patients with positive mean strength as response variable, and pipeline, lesion size and session as fixed factors and patient ID as random effect. The levels of statistical significance are denoted as: "***" for p <0.001, "**" for p <0.01 and "*" for p <0.5.

**ANOVA**	F value	p-value		**LMEM**	Estimate	std Error
				Intercept	58.7950	2.3960
Pipeline	5.0128	7.043e-3	**	CompCorLesionGS	0.3046	0.2580
				ICLesionCompCor	0.3046	0.2580
Lesion Size	1.3837	0.253		3-14	-3.3706	3.3705
				14-41	-7.3433	3.7049
				41-223	-9.1965	3.6813
Session	0.7519	0.475		3 months	-2.6515	2.4898
				1 year	-2.2968	2.2028
Pipeline:lesion size	2.8018	0.011	*	CompCorLesionGS:3-14	-0.3761	0.3614
				ICLesionCompCorGS:3-14	-0.3142	0.3614
				CompCorLesionGS:14-41	0.3162	0.3970
				ICLesionCompCorGS:14-41	0.5971	0.3970
				CompCorLesionGS:41-225	0.0961	0.3970
				ICLesionCompCorGS:41-225	0.6365	0.3970
Pipeline:session	1.6448	0.162		CompCorLesionGS:3 months	-0.3218	0.3859
				ICLesionCompCorGS:3 months	0.3228	0.3859
				CompCorLesionGS:1 year	0.0942	0.3912
				ICLesionCompCorGS:1 year	0.0979	0.3912
lesion size:session	1.0213	0.419		3-14:3 months	6.2251	3.5225
				14-41:3 months	5.1054	3.7751
				41-225:3 months	5.6670	3.8607
				3-14:1 year	4.9349	3.1362
				14-41:1 year	3.1861	3.3770
				41-225:1 year	7.2682	3.4324
pipeline:lesion size:session	0.2032	0.998		CompCorLesionGS:3-14:3 months	-0.1617	0.5473
				ICLesionCompCorGS:3-14:3 months	-0.1326	0.5473
				CompCorLesionGS:14-41:3 months	0.1193	0.5900
				ICLesionCompCorGS:14-41:3 months	-0.1244	0.5900
				CompCorLesionGS:41-225:3 months	-0.2649	0.5961
				ICLesionCompCorGS:41-225:3 months	-0.4501	0.5961
				CompCorLesionGS:3-14:1 year	-0.1413	0.5597
				ICLesionCompCorGS:3-14:1 year	-0.1789	0.5597
				CompCorLesionGS:14-41:1 year	-0.1671	0.6064
				ICLesionCompCorGS:14-41:1 year	-0.3839	0.6064
				CompCorLesionGS:41-225:1 year	-0.5144	0.6064
				ICLesionCompCorGS:41-225:1year	-0.8301	0.6064

Table S2: Results of ANOVA and LMEM of ischemic stroke patients with negative Functional Connectivity Contrast as response variable, and pipeline, lesion size and session as fixed factors and patient ID as random effect. The levels of statistical significance are denoted as: "***" for p <0.001, "**" for p <0.01 and "*" for p <0.5.

ANOVA	F value	p-value		LMEM	Estimate	std Error
				Intercept	-19.8106	3.3529
Pipeline	9.0362	1.539e-5	***	CompCorLesionGS	0.4063	2.2604
				ICLesionCompCor	0.4062	2.2604
Lesion Size	0.8092	0.493		3-14	3.5520	3.9967
				14-41	-4.8621	3.9967
				41-223	-2.0524	3.9839
Session	2.4920	0.092	.	3 months	1.6873	3.1305
				1 year	1.5772	3.3424
Pipeline:lesion size	1.7375	0.112		CompCorLesionGS:3-14	0.0771	2.6946
				ICLesionCompCorGS:3-14	0.4033	2.6946
				CompCorLesionGS:14-41	3.5161	2.6946
				ICLesionCompCorGS:14-41	4.4540	2.6946
				CompCorLesionGS:41-225	2.4029	2.6946
				ICLesionCompCorGS:41-225	5.0089	2.6946
Pipeline:session	0.2273	0.923		CompCorLesionGS:3 months	0.4148	3.3089
				ICLesionCompCorGS:3 months	0.7202	3.3089
				CompCorLesionGS:1 year	0.0075	3.4528
				ICLesionCompCorGS:1 year	0.0075	3.4528
lesion size:session	1.0017	0.434		3-14:3 months	-2.9690	3.7976
				14-41:3 months	3.5341	3.7460
				41-225:3 months	1.9257	3.7853
				3-14:1 year	-1.8908	3.9919
				14-41:1 year	1.2393	3.9907
				41-225:1 year	4.5798	3.9999
pipeline:lesion size:session	0.4401	0.946		CompCorLesionGS:3-14:3 months	-0.1150	4.0024
				ICLesionCompCorGS:3-14:3 months	-0.1937	4.0024
				CompCorLesionGS:14-41:3 months	-2.9015	3.9566
				ICLesionCompCorGS:14-41:3 months	-3.5203	3.9566
				CompCorLesionGS:41-225:3 months	-1.9613	3.9781
				ICLesionCompCorGS:41-225:3 months	-3.4441	3.9781
				CompCorLesionGS:3-14:1 year	-0.2801	4.1222
				ICLesionCompCorGS:3-14:1 year	-0.4990	4.1222
				CompCorLesionGS:14-41:1 year	0.5964	4.1222
				ICLesionCompCorGS:14-41:1 year	0.9887	4.1222
				CompCorLesionGS:41-225:1 year	-1.9968	4.1222
				ICLesionCompCorGS:41-225:1year	-3.9385	4.1222

Table S3: Results of ANOVA and LMEM of ischemic stroke patients with negative mean strength as response variable, and pipeline, lesion size and session as fixed factors and patient ID as random effect. The levels of statistical significance are denoted as: "***" for p <0.001, "**" for p <0.01 and "*" for p <0.5.

ANOVA	F value	p-value		LMEM	Estimate	std Error
				Intercept	26.9218	5.39110
Pipeline	11.4742	7.502e-5	***	CompCorLesionGS	0.0501	1.0544
				ICLesionCompCor	-0.0470	1.0544
Lesion Size	0.0362	0.853		41-223	-0.2269	7.5857
Session	3.3203	0.077		3 months	4.6154	3.0640
				1 year	3.6464	3.1164
Pipeline:lesion size	3.0175	0.058		CompCorLesionGS:41-223	-0.6448	1.4276
				ICLesionCompCorGS:41-223	-1.0500	1.4276
Pipeline:session	1.8713	0.130		CompCorLesionGS:3 months	-0.8076	1.5816
				ICLesionCompCorGS:3 months	-1.6749	1.5816
				CompCorLesionGS:1 year	-1.1046	1.7218
				ICLesionCompCorGS:1 year	-1.3351	1.7218
lesion size:session	0.2504	0.784		41-225:3 months	1.1141	4.0650
				41-225:1 year	-0.0430	4.2079
pipeline:lesion size:session	0.2351	0.917		CompCorLesionGS:41-225:3 months	-0.9027	2.0867
				ICLesionCompCorGS:41-225:3 months	-1.8259	2.0867
				CompCorLesionGS:41-225:1 year	-1.0905	2.2367
				ICLesionCompCorGS:41-225:1 year	-1.6308	2.2367

Table S4: Results of ANOVA and LMEM of hemorrhagic stroke patients with positive mean strength as response variable, and pipeline, lesion size and session as fixed factors and patient ID as random effect. The levels of statistical significance are denoted as: "***" for p <0.001, "**" for p <0.01 and "*" for p <0.5.

ANOVA	F value	p-value		LMEM	Estimate	std Error
				Intercept	-14.5577	2.0648
Pipeline	4.3174	0.019	*	CompCorLesionGS	0.5023	0.8844
				ICLesionCompCor	0.3794	0.8844
Lesion Size	0.0864	0.775		41-223	-2.5490	2.8247
Session	1.5881	0.257		3 months	-3.5775	2.2173
				1 year	2.2173	3.1147
Pipeline:lesion size	1.9017	0.161		CompCorLesionGS:41-223	-0.1448	1.1974
				ICLesionCompCorGS:41-223	0.7640	1.1974
Pipeline:session	0.2174	0.927		CompCorLesionGS:3 months	0.0288	1.3266
				ICLesionCompCorGS:3 months	0.2800	1.3266
				CompCorLesionGS:1 year	-0.5368	1.4442
				ICLesionCompCorGS:1 year	-1.3351	1.4442
lesion size:session	0.4248	0.666		41-225:3 months	1.8227	2.8503
				41-225:1 year	1.7251	3.9526
pipeline:lesion size:session	0.3827	0.820		CompCorLesionGS:41-225:3 months	0.4213	1.7502
				ICLesionCompCorGS:41-225:3 months	0.7405	1.7502
				CompCorLesionGS:41-225:1 year	2.1571	1.8760
				ICLesionCompCorGS:41-225:1 year	1.3922	1.8760

Table S5: Results of ANOVA and LMEM of hemorrhagic stroke patients with negative mean strength as response variable, and pipeline, lesion size and session as fixed factors and patient ID as random effect. The levels of statistical significance are denoted as: "***" for p <0.001, "**" for p <0.01 and "*" for p <0.5.

ANOVA	F value	p-value	LMEM	Estimate	std Error
			Intercept	60.1381	14.8545
Pipeline	0.4251	0.656	CompCorLesionGS	-0.4733	1.2520
			ICLesionCompCor	0.6546	1.2520
Lesion Size	0.4084	0.751	41-223	-9.2708	14.9188
Session	0.9108	0.438	3 months	5.7860	5.4664
			1 year	6.9413	5.6356
Pipeline:lesion size	0.9800	0.447	CompCorLesionGS:41-223	0.9949	1.1712
			ICLesionCompCorGS:41-223	0.9409	1.1712
Pipeline:session	0.3377	0.851	CompCorLesionGS:3 months	0.2351	0.6260
			ICLesionCompCorGS:3 months	-0.8928	0.6260
			CompCorLesionGS:1 year	0.3032	0.6566
			ICLesionCompCorGS:1 year	-1.3059	0.6566
lesion size:session	0.9929	0.461	41-225:3 months	-7.6357	7.8266
			41-225:1 year	-8.9869	8.0144
pipeline:lesion size:session	1.4190	0.208	CompCorLesionGS:41-225:3 months	-1.1106	0.8694
			ICLesionCompCorGS:41-225:3 months	-0.0041	0.8694
			CompCorLesionGS:41-225:1 year	-1.4005	0.9133
			ICLesionCompCorGS:41-225:1 year	0.6532	0.9133

Table S6: Results of ANOVA and LMEM of hemorrhagic stroke patients with FCC as response variable, and pipeline, lesion size and session as fixed factors and patient ID as random effect. The levels of statistical significance are denoted as: "***" for p <0.001, "**" for p <0.01 and "*" for p <0.5.

References

Elizabeth Bates, Stephen M. Wilson, Ayse Pinar Saygin, Frederic Dick, Martin I. Sereno, Robert T. Knight, and Nina F. Dronkers. Voxel-based lesion–symptom mapping. *Nature Neuroscience*, 6(5):448–450, May 2003. ISSN 1546-1726. doi: 10.1038/nn1050.

Rastko Ciric, Daniel H. Wolf, Jonathan D. Power, David R. Roalf, Graham L. Baum, Kosha Ruparel, Russell T. Shinohara, Mark A. Elliott, Simon B. Eickhoff, Christos Davatzikos, Ruben C. Gur, Raquel E. Gur, Danielle S. Bassett, and Theodore D. Satterthwaite. Benchmarking of participant-level confound regression strategies for the control of motion artifact in studies of functional connectivity. *NeuroImage*, 154:174–187, July 2017. ISSN 1053-8119. doi: 10.1016/j.neuroimage.2017.03.020.

Jingwei Li, Taylor Bolt, Danilo Bzdok, Jason S. Nomi, B. T. Thomas Yeo, R. Nathan Spreng, and Lucina Q. Uddin. Topography and behavioral relevance of the global signal in the human brain. *Scientific Reports*, 9(1):14286, October 2019. ISSN 2045-2322. doi: 10.1038/s41598-019-50750-8.
